# From Antifungal Potency to Fruit Protection: Infection-Site Bioavailability and Delivery Constraints in Antifungal Edible Coatings

**DOI:** 10.3390/foods15193408

**Published:** 2026-09-24

**Authors:** Usman Rasheed, Yingxi Mo, Qurat Ul Ain, Bin Shan, Yongsen Chen, Ji Zhang, Ganlin Chen

**Affiliations:** 1 Guangxi Key Laboratory of Quality and Safety Control for Subtropical Fruits, Guangxi Subtropical Crops Research Institute, Guangxi Academy of Agricultural Sciences, Nanning 530001, China; rasheus@outlook.com (U.R.); moyingxi29@163.com (Y.M.); zzbj219@163.com (B.S.); cyongsen@163.com (Y.C.); zhangji369369@gxaas.net (J.Z.); 2 Key Laboratory of Quality and Safety Control for Subtropical Fruit and Vegetable, Ministry of Agriculture and Rural Affairs, Nanning 530001, China; 3 National Key Laboratory of Non-Food Biomass Energy Technology, Guangxi Key Laboratory of Bio-Refinery, Institute of Eco-Environmental Research, Guangxi Academy of Sciences, 98 Daling Road, Nanning 530007, China; qurat390@gmail.com; 4 Guangxi Zhuang Autonomous Region Engineering Research Center of Green and Efficient Development for Mango Industry, Nanning 530001, China

**Keywords:** postharvest anthracnose, *Colletotrichum* spp., antifungal edible coatings, infection-site bioavailability, cuticular transport, storage trajectory

## Abstract

Postharvest anthracnose caused by *Colletotrichum* spp. remains a constraint to the storage and marketing of tropical and subtropical fruits. Edible antifungal coatings carrying biopolymers and antifungal agents have received considerable attention as alternatives to conventional fungicides. Previous research has demonstrated the potential of biopolymers, essential oils, biocontrol agents, nanoemulsions, Pickering emulsions, multilayer coatings, and delivery systems. However, strong inhibition of mycelial growth or conidial germination in vitro does not consistently translate into equivalent disease control on fruit, indicating that intrinsic antifungal potency alone is insufficient to explain postharvest performance. *Colletotrichum* conidia are initially exposed at the fruit surface, but appressorium formation, cuticle penetration, and quiescent colonization progressively separate the pathogen from surface-applied actives. Meanwhile, fruit cuticles vary among hosts and cultivars and continue changing during storage. Temperature, relative humidity, condensation, ripening, and handling further influence infection and coating properties, while active physicochemical processes determine antifungal retention, partitioning, volatilization, and release. This review examines and compares edible antifungal coatings through infection-site bioavailability, integrating pathogen stage, fruit-surface transport, storage trajectory, active properties, and coating architecture to explain the in vitro–to-in situ translation gap and define context-matched design and measurement priorities.

## 1. Introduction

Postharvest anthracnose caused by *Colletotrichum* spp. is a major constraint to the storage and marketing of tropical and subtropical fruits. Infection is frequently established before harvest and may remain quiescent until fruit ripening, when expanding brown-to-black, sunken lesions and, under favorable conditions, conidial masses become evident [1,2]. The disease affects a broad range of hosts, including mango, papaya, guava, avocado, banana, citrus, and strawberry. Broad estimates for developing-country production systems place postharvest losses at 30–60% for mango and 20–40% for guava [3]. For papaya, losses of up to 50% have been cited in the literature without attribution to a specific national or regional production system [4]. A study from Sri Lanka reported anthracnose-associated losses of approximately 30–40% of marketable banana fruit [5]. By contrast, the avocado estimate is geographically defined in a multi-regional Colombian study involving Hass-avocado production plots and packinghouse records; anthracnose-associated rejection reached up to 52.5% in affected production systems [6].

A central issue addressed in this review is that strong antifungal activity measured under laboratory conditions does not necessarily translate into comparable disease control on fruit. Chitosan and *Mentha piperita* essential oil mixtures strongly inhibited five *Colletotrichum* species in vitro, yet de Oliveira et al. (2017) [7] noted that these effects are not always confirmed after application to mango. In papaya, chitosan with Mentha oils produced additive or synergistic inhibition in culture, while protection varied among isolates and storage times [8]. A nanostructured chitosan-thyme coating completely inhibited *C. gloeosporioides* mycelial growth and conidial germination in vitro, whereas disease incidence on Hass avocado was recorded as 54.0%, compared with 84.6% in uncoated fruit [9]. By contrast, chitosan containing 400 μL L^−1^ thyme oil provided effective control of mango anthracnose, consistent with the complete inhibition of fungal growth observed in vitro at the same concentration [10]. Table 1 summarizes 20 studies comparing laboratory antifungal activity with disease control on fruit across different coating matrices, active compounds, fruit hosts, *Colletotrichum* species, and storage conditions. Fifteen studies reported complete in vitro inhibition, whereas the corresponding disease protection on fruit remained incomplete, with calculated efficacy gaps ranging from approximately 15% to 100%. The magnitude of this discrepancy varied considerably among host–coating systems; for example, the same chitosan–*Cymbopogon citratus* essential-oil treatment showing complete in vitro inhibition provided 63.5%, 55.7%, and 85% disease control in guava, mango, and papaya, respectively. These comparisons indicate a recurring efficacy translation gap, although its numerical magnitude should be interpreted cautiously because the studies used different disease endpoints and experimental conditions.

This discrepancy is referred to here as the in vitro–to-in situ fruit translation gap, reflecting the observation that antifungal effectiveness under laboratory conditions does not always correspond to the level of disease control achieved following application on fruit. Here, infection-site bioavailability is defined operationally as the amount or concentration of antifungal active present in a biologically accessible form at the pathogen’s relevant anatomical location during the infection-relevant time window. Retention, available fraction, partitioning, transport, and persistence are therefore treated as determinants of this local exposure rather than as equivalent measures of bioavailability. Interpretation of antifungal edible coating performance is also influenced by the endpoint assessed. Mycelial growth inhibition, conidial germination, lesion diameter, disease incidence, disease severity, and decay represent distinct biological and pathological outcomes, and their reported percentage values are not necessarily directly comparable. When different endpoints are used, the available evidence may therefore be more appropriately interpreted qualitatively rather than expressed as a numerical difference in efficacy. Recognizing these distinctions can help differentiate variation arising from experimental design and outcome measurement from differences in biological performance. Accordingly, three levels of evidence are distinguished throughout this review. In vitro inhibition of mycelial growth or conidial germination demonstrates intrinsic antifungal activity under the assay conditions, but does not by itself demonstrate suppression of disease development on intact fruit. On-fruit disease reduction provides evidence of biological efficacy at the fruit level, but does not establish that the active compound reached a quiescent cuticular or subcuticular infection site. Demonstration of an integrated host–pathogen–coating mechanism would require additional spatial and temporal evidence linking coating-derived active availability at the infection site with pathogen and/or host responses. These levels of evidence are therefore treated separately in the following sections.

In this context, four interacting constraints can separate nominal antifungal loading from biologically effective exposure. First, pathogen stage and location change during infection. *Colletotrichum* can form appressoria, penetrate the cuticle, and remain quiescent beneath it or within the first cell layers until ripening increases host susceptibility [1]. Second, the fruit surface is a variable interface. Fruit cuticles differ chemically and biophysically among species and cultivars and continue changing after harvest in response to external conditions and treatments [11]. Third, storage temperature, humidity, duration, and ripening state determine the required protection period, while quiescent infections may become symptomatic only after prolonged storage or during shelf life [1]. Fourth, natural antifungals, especially essential oils, can exhibit high volatility, poor water solubility, instability, and limited matrix compatibility, restricting persistence and delivery [12,13].

Fruit coating architecture therefore governs active retention, dispersion, and release during storage. Encapsulation of *Angelica archangelica* essential oil in a chitosan nanoemulsion produced biphasic controlled release and sustained protection of grapes against *Botrytis cinerea* for 30 days, providing mechanistic evidence that architecture can alter persistence outside the *Colletotrichum* pathosystem [14]. In mango, thyme-oil nanoemulsion delayed anthracnose during 30 days at 10 °C, whereas its combination with D-limonene provided no additional preservation benefit [15]. In strawberry, the formulation with the strongest in vitro antifungal activity did not provide the best performance on fruit, while slower thyme-oil release from a chitosan–pullulan formulation was associated with better preservation [16].

Existing reviews have already covered edible-coating materials, preservation functions, advanced formulations, and postharvest quality maintenance [12], while Romanazzi and Moumni (2022) [17] specifically synthesized chitosan and related coatings for decay control. Essential oils against *Colletotrichum* have also been reviewed across conventional coatings, emulsions, and encapsulation approaches [18]. The unresolved issue is therefore not a lack of technologies or antifungal candidates. Rather, these bodies of evidence do not provide a unified basis for selecting coating architecture from the combined requirements imposed by infection biology, the fruit interface, storage conditions, and active physicochemistry. This limits mechanistic interpretation of why similar antifungal agents can perform differently among fruits and storage scenarios. To address this gap, this review examines antifungal edible coatings from the perspective that no single architecture is universally optimal across diverse postharvest disease systems. Suitability is conditional on the pathogen, fruit surface, storage trajectory, and properties of the active. It analyzes the biological and physicochemical limitations to determine the necessary exposure for control and compares different coating structures to demonstrate that exposure. In this review, evidence from direct fruit-coating studies is given primary emphasis, while findings from packaging systems, non-*Colletotrichum* pathosystems, and model delivery studies are incorporated where they provide relevant mechanistic insight. This review suggests that rational design must begin by identifying where and when the pathogen must be controlled and defining the fruit barrier and storage conditions separating the applied antifungal from that target.

### Review Scope and Literature Selection

This article is a critical narrative review supported by a structured literature search. We searched Web of Science Core Collection and Scopus, using Google Scholar for supplementary targeted searches and backward and forward citation tracking. The final search was conducted on 20 September 2026, with no lower publication-year restriction. Searches were restricted to English-language, peer-reviewed journal articles and reviews; peer-reviewed early-access articles were also eligible. Conference abstracts and proceedings, editorials, letters, book chapters, theses or dissertations, patents, preprints, and non-English publications were excluded. The complete database-specific search strings are provided in Supplementary Table S1.

The search was organized around three evidence domains: (i) antifungal edible coatings applied to postharvest fruit, with priority given to studies involving *Colletotrichum* spp. or anthracnose; (ii) mechanistic evidence concerning pathogen developmental stage, fruit cuticle and wax properties, coating adhesion, partitioning, permeability, active release, and storage-dependent changes; and (iii) adjacent delivery or analytical studies used only when they addressed a specific mechanistic or measurement gap relevant to the review. Studies were included when they addressed at least one of the following: on-fruit disease control; pathogen developmental stage or anatomical location; coating composition, architecture, or active release; fruit-surface or cuticular transport; storage-dependent changes in the coating or fruit interface; or methods relevant to measuring transport and local exposure. Studies lacking direct relevance to postharvest fruit disease, antifungal coating or delivery, fruit-surface transport, or the mechanistic comparisons developed in this review were excluded. Duplicate records were removed before title/abstract screening and subsequent full-text assessment. Because this article is a critical narrative review rather than a systematic review, the structured search was used to improve transparency and coverage rather than to claim exhaustive retrieval or support meta-analytic pooling.

**Table 1:** Comparative in vitro and in situ fruit performance of antifungal edible coatings against postharvest anthracnose across fruit hosts.

Host Fruit	Coating Matrix	Antifungal Agent/Form	Application Method	Pathogen	In Vitro Efficacy	In Situ Fruit Efficacy	Storage Condition (T and RH)	Storage Time (Days)	In Vitro–In Situ Efficacy Gap (%)	Reference
Mango	Chitosan	*Mentha piperita* essential oil	Dip coating (5 min)	*C. asianum* CMM 4057	100% growth inhibition (CHI 5 mg/mL + MPEO 1.25 μL/mL)	~53%	25 °C; RH NR	15	47	[7]
Avocado	Nanostructured chitosan coating	Thyme essential oil (nanoparticle formulation)	Dip coating (30 s)	*C. gloeosporioides*	100% growth inhibition	63%	27 ± 2 °C; RH NR	6	37	[9]
Guava, mango, papaya	Chitosan	*Cymbopogon citratus* essential oil (free EO)	Dip coating (1 min)	*C. asianum*	100% growth inhibition	Guava: 63.5% Mango: 55.7% Papaya: 85%	25 °C, RH NR	12	15–44.3	[19]
Papaya	Chitosan coating	*Mentha piperita* L. essential oil (free EO)	Dip coating (1 min)	*C. gloeosporioides CMM 24*	100% growth inhibition	38%	25 ± 0.5 °C; RH NR	10	62	[8]
Citrus	Chitosan	Sodium ethylparaben (synthetic additive)	Brush coating	*C. gloeosporioides*	100% growth inhibition	Nadorcott mandarins: ~53%, Alencia oranges: ~10%	25 °C; 95% RH	7	47–90	[20]
Mango and papaya	Amylose starch	Carvacrol + thymol (free phenolic compounds)	Dip coating (2 min)	*C. gloeosporioides*	100% growth inhibition (MIC 1500 for both carvacrol and thymol at pH 5)	Mango 52%, Papaya 32%	20 °C; RH NR	18	48–48	[21]
Mango	Chitosan coating	Thyme oil (free EO)	Dip coating (2 min)	*C. gloeosporioides*	100% growth inhibition (400 μL L^−1^ dose)	~77.27%	12 °C; RH 80–85% for 28 d and 5 d at 25 °C	33	23	[10]
Papaya	Chitosan coating	Mint essential oil (free EO)	Dip coating (5 min)	*C. gloeosporioides*	100% growth inhibition	0% (4 day delay in disease progression vs. control)	12 °C; RH NR	20	100	[4]
Mango	Nanoemulsion coating	D-limonene + thyme oil (nanoemulsion)	Dip coating (2 min)	*C. gloeosporioides*	49% growth inhibition	~20%	10 °C; RH NR	30	59	[15]
Mango	Chitosan coating	Iturin A (antifungal lipopeptide)	Dip coating (3 min)	*C. gloeosporioides*	~75% growth inhibition (CTS-0.12IA)	~62%	12 °C; RH NR	20	17	[22]
Tomato	Chitosan coating	Thyme essential oil (nanoemulsion)	Dip coating (1 min)	*C. gloeosporioides*	100% growth inhibition (Nano-(T)-EO-CS)	~50%	10 °C; RH 85%, then 18 °C shelf simulation	14 d cold + 3 d shelf	50	[23]
Mango	Sodium alginate	Mixed lactic acid bacteria (microbial biocontrol)	Dip coating (1 min)	*C. fruticola*	100% growth inhibition	Mango 77.80 Guava 77.5	25 °C; RH 85%	15	22	[3]
Avocado	Chitosan	Thyme essential oil (free EO)	Dip coating	*C. gloeosporioides*	100% growth inhibition	80%	20 °C, RH NR	5	20	[24]
Bell pepper	Gelatin	Green and brown propolis (natural extract)	Dip coating (30 s)	*C. scovillei*	100% growth inhibition	59.4–65.6%	25 °C, 12 h photoperiod, RH NR	6	34–40	[25]
Avocado	Sodium alginate	*Meyerozyma caribbica* (microbial biocontrol yeast)	Dip coating	*C. gloeosporioides*	99.6% growth inhibition	53%	25 °C; RH 75%	15	47	[26]
Mango	Zein nanoparticles	Curcumin (nanoparticle formulation)	Dip coating (1 min)	*C. gloeosporioide*	~73% growth inhibition	~60%	32 °C; RH NR	9	17	[27]
Strawberry	Carboxymethylcellulose	*Lippia sidoides* essential oil (free EO)	Dip coating (2 min)	*C. acutatum*	100% growth inhibition	~22%	25 °C, 95% RH	7	72	[28]
Banana	10% TWEEN-80	Thyme essential oil (TWEEN-80 dispersion)	Spray coating	*C. musae*	100% growth inhibition	~31%	13 °C; RH 80% for 21 d, and 7 d at 20 °C; RH 85%	30	69	[29]
Strawberry	Carboxymethylcellulose	*Eucalyptus staigeriana* essential oil (free EO)	NR	*C. acutatum*	97.4% growth inhibition	~9%	25 °C; 95% RH; 12 h photoperiod	7	90	[30]
Avocado	Carboxymethylcellulose	*Lippia sidoides* essential oil (free EO)	Dip coating (2 min)	*C. gloeosporioide*	100% growth inhibition	44%	10 °C; RH 85%	28	54	[31]

Note: NR, not reported; EO, essential oil; RH, relative humidity. The in vitro–in situ efficacy gap represents the difference between in vitro antifungal inhibition and on-fruit disease control when comparable percentage metrics were available.

## 2. The Pathogen–Fruit Interface as the Primary Design Constraint

*Colletotrichum* infection presents a dynamic delivery challenge. Conidia are initially accessible on the fruit surface, whereas subsequent appressorium differentiation, cuticle penetration, and superficial quiescent colonization progressively reduce the pathogen’s direct exposure to surface-applied antifungal agents. Accordingly, assays against surface-exposed conidia primarily characterize antifungal activity during the early, surface-accessible stage and, by themselves, do not demonstrate that the applied antifungal reaches the pathogen after cuticle penetration [1,32,33]. The intervening biological surface is equally variable. The cuticle is a chemically stratified composite whose geometry, microchemistry, wax deposition, and natural openings differ among cultivars and continue to change after harvest [34,35,36]. Target accessibility is thus a joint property of pathogen position and interface state. This section resolves that joint dependence into six spatial design variables, including coverage, adhesion and continuity, available fraction, partitioning and penetration, target-site persistence, and surface presentation.

### 2.1. Locating the Biological Target Across the Infection Sequence

#### 2.1.1. Surface Deposition and Germination

Conidia deposited on intact fruit are the most directly accessible target. In the tomato pathosystem, germination on unripe fruit cuticle culminated in appressorial differentiation at approximately 19 h post-inoculation [32]. Within this window, a surface-applied coating contacts the spores without requiring transcuticular transport, and performance depends only on whether an inhibitory concentration is present in the vicinity of conidia. The antifungal coating design requirement is therefore early availability combined with sufficiently uniform coverage of likely deposition sites, achieved within a short and host-dependent surface-accessible window [32,33].

#### 2.1.2. Appressorium Formation and Penetration

Appressorium differentiation alters both fungal physiology and target accessibility. Wang et al. (2018) [33] reported extensive transcriptional differences between *C. gloeosporioides* appressoria and vegetative hyphae, establishing the appressorium as a distinct physiological state. This comparison was made between fungal structures and carries no information about position within fruit tissue. Direct evidence in the mango pathosystem comes from gene disruption. *LAC1* mutants defective in melanized appressorium formation lost pathogenicity on unwounded leaves and fruits while retaining reduced virulence on wounded tissue [37], establishing melanized appressorium formation as a genetic requirement for the intact-tissue route rather than a morphological stage. In tomato, the appressorium and the emergence of non-infectious hyphae were found to be confined to the cuticle interface [32]. Once penetration begins, direct antifungal action at the infection site would require an externally applied active to remain available and reach the cuticular or subcuticular target. This requirement is mechanistically inferred from pathogen location; it has not been demonstrated by direct measurement of coating-derived antifungal transport to the infection site.

#### 2.1.3. Quiescent Infection Within the Fruit Interface

After penetration the target occupies a superficial but shielded compartment. In tomato, the quiescent stage was characterized by the development of dendritic-like structures and swollen hyphae within the fruit epidermis [32]. Described in finer spatial detail, the dendritic-like protrusions form within the cuticle itself, whereas the swollen hyphae colonize the first epidermal cell layer [38]. The quiescent target is therefore distributed across two adjacent compartments rather than occupying a single plane. For a surface-applied antifungal to act directly on such a target, it would need to reach the cuticular and, where relevant, outer epidermal compartment. This is a proposed delivery requirement rather than an experimentally demonstrated coating–pathogen interaction. Prusky et al. (2026) [1] described comparable superficial quiescent persistence across postharvest pathogens. These observations do not establish a universal infection depth for mango or other fruits. Coatings intended for this stage would therefore require persistence and a plausible interfacial or transcuticular transport pathway, whose operation must be verified experimentally. These observations do not establish a universal depth for mango or other fruits, and that restraint matters because mango anthracnose is caused by several *Colletotrichum* species whose relative importance differs between tissues and regions, with *C. asianum* prominent among fruit isolates [39]. Coatings designed for this stage consequently require persistence and, if the target is below the surface, should involve a mechanistically plausible interfacial or transcuticular pathway.

#### 2.1.4. Exit from Quiescence and the Variable Target Interval

Quiescence has been documented in mango, avocado, banana, papaya, strawberry, and citrus [1]. In each host, early fruit defenses restrict fungal growth, whereas symptom development is delayed until ripening weakens fruit resistance and softens tissues. The anatomical sequence is conserved, but the duration of quiescence differs. In climacteric fruits such as mango, avocado, and banana, ethylene-driven ripening extends the pre-necrotrophic interval, during which the pathogen remains below the cuticle [1,40]. In non-climacteric hosts such as strawberry and citrus, the window is shorter and governed by different quiescence-exit signals [1,41]. On mature green tomato, quiescence ended at approximately 10 d post-inoculation, when hyphae extended from the swollen hyphal structures and necrotrophic hyphae appeared, whereas on ripe fruit the necrotrophic stage was already established by 48 h post-inoculation [32]. Supported transition factors include increased accessibility of disassembled cell-wall substrates, weakening of inducible defense, decline of preformed antifungal compounds, and pathogen-associated modification of the local environment, including the alkalization reported during quiescence [1,32]. Sunlight-exposed red mango skin accumulated higher levels of glycosylated flavonols, glycosylated anthocyanins, and mangiferin and was more resistant to *C. gloeosporioides* than green fruit [42]. Antifungal availability must therefore persist until quiescence ends, over an interval defined by fruit physiological state and preharvest history rather than by a fixed duration. Establishing where the pathogen resides is necessary but not sufficient. The barrier separating an externally applied coating from that position must be defined with equal precision. Figure 1 illustrates four biologically distinct coating–infection scenarios and the corresponding differences in pathogen location and exposure route.

#### 2.1.5. Coupling Fungal Infection Biology with Coating Function

The relevant mechanistic question is therefore not simply the host–pathogen interaction, but how an applied coating may influence that interaction. During surface germination, the coating can act directly because conidia remain accessible, and efficacy depends primarily on local active-compound availability. Appressorium differentiation introduces both a physiological and spatial transition: transcriptional reprogramming accompanies appressorium development [33], while LAC1-dependent melanization is required for full pathogenicity on intact mango tissue [37]. A coating intended to interfere with this stage must therefore deliver an active compound while the penetration structure remains accessible, rather than relying solely on activity measured against free conidia.

Chitosan should not be considered uniformly fungicidal against *Colletotrichum*. Depending on its physicochemical properties, concentration, fungal isolate, and host, its effects may include inhibition or delay of conidial germination and mycelial development together with elicitation of host defenses. A meta-analysis confirmed reductions in fungal growth and postharvest disease together with increased chitinase and β-1,3-glucanase activities in treated fruit [43], while transcriptomic analysis of the avocado–chitosan–*Colletotrichum* interaction demonstrated regulation of defense-, transport-, and signaling-related pathways [44]. Essential-oil constituents may additionally affect specific fungal targets; for example, eugenol interfered with the *C. gloeosporioides* laccase *Cglac4*, which is associated with conidial germination, appressorium formation, and pathogenicity [45]. Thus, coating components may influence both pathogen development and host defense, although direct interference of specific coating functional groups with host–pathogen signaling at the infection site remains largely unverified and represents an important mechanistic gap.

After appressorium-guided fungal penetration, coating function becomes increasingly dependent on the fruit interface. Quiescent development is regulated by host physiological state, including changes in defense capacity, phenolic composition, tissue pH, and ripening-associated metabolism [1,38,42]. At the same time, *Colletotrichum* can modify its local environment through ammonia-associated alkalinization, which has been linked to appressorium formation and pathogenicity [46,47,48,49]. These processes provide potential mechanistic cues for coating design. For example, pH-responsive carriers could couple antifungal release to infection-associated chemical changes, whereas chitosan-containing coatings may combine direct antimicrobial activity with induction of host defense responses [50]. However, direct evidence demonstrating that such coatings respond to these molecular events at the infection court remains limited. Accordingly, coating–pathogen interactions proposed for post-penetration and quiescent stages should be interpreted as mechanistic hypotheses derived from infection anatomy, host physiology, and material behavior rather than as experimentally established interactions.

During the transition to necrotrophy, fungal extracellular enzymes, including laccases, cellulases, pectinases, and pectate lyases, become increasingly relevant [46,51]. These enzymes provide a further conceptual link to degradable or enzyme-responsive delivery systems, although this strategy has not yet been directly validated for anthracnose on fruit [52,53]. Thus, the mechanistic value of a coating lies not in reproducing the known infection sequence, but in matching active release, transport, and persistence to the fungal physiological state and host microenvironment at each stage.

### 2.2. Fruit-Surface Architecture as Antifungal Delivery Constraints

#### 2.2.1. The Cuticle as a Stratified Composite Interface

Fruit cuticle comprises a cutin polyester matrix with cell-wall polysaccharides, embedded intracuticular waxes, and an outer epicuticular wax layer that may occur as crystals, platelets, or continuous films [54]. Fruit waxes contain C20–C34 very-long-chain fatty-acid (VLCFA) derivatives, including alkanes, aldehydes, alcohols, ketones, and esters, together with triterpenoids, sterols, and phenolics. Cutin is assembled mainly from C16 and C18 hydroxy- and epoxy-hydroxy fatty-acid monomers [54]. These structural and chemical differences govern coating wetting, sorption, diffusion, and retention of antifungal compounds. Heredia et al. (2024) [36] described the cuticle as an open supramolecular composite in which a cutin-rich matrix is associated with polysaccharides and incorporates waxes and phenolic constituents. Tomato is included here as a mechanistically informative fruit model because infection of tomato fruit by *C. gloeosporioides* has been experimentally characterized [32], and anthracnose control on stored tomato has also been evaluated using antifungal edible coatings [23]. This spatial heterogeneity has been resolved by confocal Raman imaging of tomato fruit cuticles, where cutin extended throughout the cuticular layer, phenolic acids were preferentially localized toward the outer region, and flavonoids exhibited an increasing gradient toward the inner surface. The relative distribution of these components also varied with fruit development [35]. Because these depth-resolved microchemical observations were obtained from tomato, they are used here only to illustrate cuticular stratification and should not be interpreted as direct surrogates for mango or papaya fruit surfaces. Correlated multimodal imaging further relates this architecture to mechanical properties [55]. The barrier is therefore stratified along the direction of transport, so active antifungal partitioning behavior cannot be represented by a single value for the cuticle as a whole.

#### 2.2.2. Cultivar-Dependent Cuticular Architecture and Postharvest Cuticle Dynamics

Fruit hosts and cultivars differ in cuticle thickness, wax crystallinity, polarity, triterpenoid load, cutin chemistry, lenticels, microcracks, and storage-induced remodeling. Camacho-Vázquez et al. (2019) [34] demonstrated substantial cultivar-dependent differences in epicuticular wax deposition, lenticel geometry, cuticle thickening, transpiration, and postharvest deterioration. Cuticle properties also remain dynamic after harvest: postharvest environmental treatments can alter cutin and wax composition and the expression of cuticle-related genes [56]. Thus, the surface onto which a coating is initially deposited should not be regarded as a static interface throughout storage.

Evidence from coated fruits further indicates that coating performance depends on direct interaction with this native surface. Cai et al. (2026) [57] showed that adhesion of a chitosan-based coating to highly waxy fruit surfaces could be strengthened through a Ca–gallic acid network, with molecular simulations supporting stronger van der Waals and electrostatic interactions between the coating and epicuticular-wax components. This demonstrates that cuticular wax chemistry can directly determine coating adhesion rather than acting only as a passive transport barrier. At a larger structural scale, postharvest wax coating of apple fruit maintained a comparatively compact epidermal surface and reduced the development of surface cracking during storage relative to uncoated fruit [58]. In mango, the application method also altered coating continuity, with dipping and electrostatic spraying producing more complete films than brushing, which generated tracks, cracks, and pores [59].

Host-specific evidence from major anthracnose hosts supports several of these interface concepts. In mango, cuticle architecture, epicuticular-wax deposition, lenticel abundance, and postharvest remodeling differ substantially among cultivars [34]. Wu et al. (2023) [60] further showed cultivar-dependent changes in mango epicuticular-wax content, composition, and crystal morphology and found that experimental disruption of the wax layer increased early *C. gloeosporioides* incidence and/or disease index depending on cultivar. Coating–surface behavior has also been examined directly on mango: sequential chitosan–alginate layers altered surface wettability [61], while application method affected film continuity [59]. In papaya, postharvest cuticle morphology and wax/cutin composition are likewise cultivar- and temperature-dependent [62], while a recent anthracnose study associated greater resistance with a thicker cuticle, lower stomatal density, and more robust surface-associated defenses [63]. However, although antifungal coatings have been evaluated extensively on papaya [4,8], direct measurements linking coating adhesion strength or cuticular transport to antifungal concentration at a subcuticular *Colletotrichum* infection site remain unavailable. Thus, host-specific evidence supports the importance of the native surface barrier, but the mechanistic link between coating adhesion and infection-site delivery remains unresolved in both mango and papaya.

At the molecular level, the coating–cuticle interaction also depends on the chemical structure and ionization state of the coating components. Chitosan is a linear β-(1→4)-linked polysaccharide containing D-glucosamine and N-acetyl-D-glucosamine units. Under the mildly acidic conditions commonly used to prepare chitosan coatings, its primary amino groups become protonated (–NH_3_^+^), giving the polymer a pH-dependent cationic character; consequently, pH, molecular weight, and degree of deacetylation influence its charge density, solubility, film formation, and antimicrobial behavior [50]. However, the intact fruit surface should not be regarded as a uniformly anionic substrate because the outer cuticle is dominated by cutin and hydrophobic epicuticular and intracuticular waxes [36,54]. Chitosan adhesion to this interface can therefore involve a combination of hydrogen bonding, van der Waals forces, and, where ionizable surface groups are accessible, electrostatic interactions. This interpretation is supported by recent molecular-dynamics analysis of a modified chitosan coating on blueberry wax, which identified combined van der Waals and electrostatic contributions to coating–wax adhesion [57].

Essential oils should not be regarded as chemically uniform antifungal ingredients. They are multicomponent mixtures whose composition depends on botanical source and chemotype and may include hydrocarbon monoterpenes, oxygenated monoterpenes such as alcohols, aldehydes, ketones, phenolic monoterpenoids, sesquiterpenes, and phenylpropanoid-derived compounds [18,64,65]. For example, limonene is a hydrocarbon monoterpene; thymol and carvacrol are phenolic monoterpenoids; menthol is a monoterpene alcohol; citral comprises oxygenated monoterpene aldehydes; and eugenol and cinnamaldehyde are phenylpropanoid-derived aromatic compounds. These chemical families differ in polarity, vapor pressure, hydrogen-bonding capacity, and chemical reactivity and can therefore differ in polymer-matrix affinity, partitioning into cuticular waxes, volatilization, release kinetics, and antifungal activity. Moreover, whole-oil activity may reflect additive, synergistic, or antagonistic interactions among constituents rather than the activity of the predominant component alone [18]. Consequently, total essential-oil loading is insufficient to define an antifungal formulation; botanical source, chemotype, and major constituent profile should also be considered when interpreting coating performance. Although hydrophobic hydrocarbon terpenes may preferentially associate with lipid-rich domains and oxygenated and phenolic constituents can exhibit different intermolecular interactions, such behavior remains compound- and matrix-dependent and should not be treated as a universal cuticular transport rule [36,54,64,66].

Together, these findings suggest a testable coating–cuticle hypothesis: infection-site bioavailability should depend not only on release from the coating but also on how the coating associates with epicuticular wax, spans or penetrates surface discontinuities, and remains coupled to a cuticle that changes during storage. Strong surface adhesion may improve persistence, but it may also retain a lipophilic antifungal within the outer coating–wax domain, thereby reducing transport toward a subcuticular infection site. Conversely, partial filling of microcracks or lenticels may alter both local water exchange and potential transport pathways. These possibilities remain hypotheses because direct measurements linking coating-induced changes in cuticle microstructure or chemistry with antifungal concentration at the *Colletotrichum* infection court are currently lacking. This represents an important mechanistic gap for future coated-fruit studies.

#### 2.2.3. Wetting, Spreading, Adhesion and Film Continuity

Surface contact precedes release and diffusion of the active antifungal from the coating matrix. Consequently, coating performance depends initially on wetting, spreading, adhesion, and formation of a sufficiently continuous layer on the chemically heterogeneous fruit surface. Contact angle and surface-energy measurements provide useful approaches for characterizing these interfacial properties [36]. Direct measurements on mango further support this relationship: sequential chitosan–alginate deposition produced changes in surface wettability and generated multilayer coatings closely associated with the fruit surface [61]. More recently, strengthening non-covalent interactions between chitosan-based coatings and epicuticular wax substantially improved adhesion on wax-rich fruit surfaces [57]. Application method also determines the final coating architecture; on mango, dip coating and electrostatic spraying produced relatively uniform and complete layers, whereas brushing generated tracks, cracks, and pores [59]. Formulation rheology is another determinant of deposition behavior, as a chitosan-particle-stabilized thyme-oil Pickering emulsion showed shear-thinning and elastic characteristics considered suitable for spraying [13]. Together, these studies indicate that formulation properties and fruit-surface chemistry jointly determine the coating actually established on the fruit. However, direct relationships between coating continuity or adhesion and antifungal concentration at the *Colletotrichum* infection site remain largely unquantified.

#### 2.2.4. Partitioning and Diffusion Through the Intact Cuticle

Partitioning determines where an active preferentially resides, whereas diffusion determines its movement through the available pathway. These two factors should not be collapsed into a single notion of penetration. In isolated leaf cuticles of *Prunus laurocerasus* and *Garcinia xanthochymus*, removal of the cyclic wax fraction left permeance essentially unchanged, whereas removal of the very-long-chain aliphatic fraction substantially increased permeance for both the lipophilic azoxystrobin and the hydrophilic theobromine [66]. Wax composition rather than total wax abundance is therefore the supported transport variable, and the same aliphatic fraction limited solutes at both ends of the polarity range examined; this remains leaf evidence and is not a fruit-cuticle measurement. As discussed earlier, the fruit cuticle is chemically stratified (Section 2.2.1); therefore, partitioning is additionally depth-dependent, and an actively accumulating outer phenolic or wax-rich domain is not necessarily positioned to reach a subcuticular target. Tredenick and Farquhar (2021) [67] modeled water sorption through cellulose-associated, aqueous-pore, and lipophilic contributions and identified a quantitatively important cellulose component, supporting hydration state as a variable barrier property. Neither study assigns an antifungal to a specific route in fruit. Surface retention may accordingly increase persistence while still leaving an insufficient available fraction at a shielded target.

#### 2.2.5. Microcracks, Lenticels, Wounds and Enzymatic Breach

Cuticular discontinuities create qualitatively distinct pathways for pathogen access. Fractures in the cuticle can facilitate pathogen penetration, while hydration has been associated with increased microcracking in several fruits, including mango [36]. Microcracks, wounds, and lenticels are also recognized as potential entry sites for pathogens [11], and lenticel abundance can vary substantially among cultivars [34]. Prusky et al. (2026) [1] distinguish direct penetration from wound-assisted entry and describe penetration structures associated with cutinase and lipase activity, indicating that the barrier at infection sites may differ locally from intact cuticle elsewhere on the fruit. This distinction is supported experimentally by an appressorium-defective mango mutant that was non-pathogenic on unwounded tissue but retained reduced virulence following wounding [37]. Wounding alters the infection biology itself rather than only the barrier, since *C. gloeosporioides* germinating on small wounds of unripe fruit skipped the biotrophic-like quiescent stage and colonized necrotrophically [32]. Wound models therefore test both a partly bypassed barrier and a different disease course, and cannot demonstrate transcuticular delivery of active antifungal across intact fruit.

### 2.3. Matching Pathogen Location to Required Spatial Delivery Behavior

For surface-exposed propagules, transcuticular delivery is unnecessary. The priority is uniform coverage and early availability during the short interval preceding appressorial differentiation. Rapid release is advantageous only insofar as it serves this early target rather than as a generally superior release profile. Inhibition of germination or appressorium formation at the surface does not establish control of infections that have already penetrated [32,33]. At the appressorium and penetration stage, adhesion, film continuity, and sustained local availability become critical. Because melanized appressorium formation is genetically required for infection of intact mango tissue, this stage represents a mechanistically important intervention point, as disruption of functional appressorium formation prevents the intact-tissue infection route [37]. However, this inference is based on a mutant phenotype and does not establish the superiority of any coating architecture. For quiescent superficial infection, effective control depends on target-site persistence and sustained availability of the antifungal agent at the cuticle–epidermal interface. The relevant target region extends across both the cuticular and outer epidermal compartments rather than being confined to a single surface plane. The extent of required transcuticular transport is likely to depend on host species and infection site, with tomato currently providing the most spatially resolved example. Persistence is therefore better defined relative to fruit maturity and storage conditions than as a fixed duration, because the quiescent phase varies with physiological stage [1,32,38].

Wound-exposed tissue constitutes a separate evidence category. The intact cuticle is partly bypassed, and the appressorium-defective mutant that lost pathogenicity on unwounded mango retained reduced virulence on wounded tissue [37]. Wounding also redirects the infection biology toward direct necrotrophic colonization without an intervening quiescent phase [32]. Efficacy observed in wound models can therefore overestimate relevance to intact-fruit delivery on two counts, and wound and intact-fruit evidence should not be pooled.

Active physicochemistry determines which coating function is limiting within each target state. Volatility governs whether the active must be retained or released into a headspace: Pickering encapsulation raised thyme oil headspace concentration from 0.21 to 1.86 g L^−1^ after two gas-release events, indicating that encapsulation modified volatile availability rather than merely retaining the active [13]. Polarity and matrix affinity act principally through wax composition, since the very-long-chain aliphatic fraction limited both lipophilic and hydrophilic model solutes [66], while chemical stratification of the fruit cuticle makes the relevant partitioning depth-dependent [35]. Ionization is expected to respond to local pH, so the alkalization reported during quiescence may alter the effective partitioning of ionizable actives, although this has not been measured for any antifungal–fruit pair [32].

Six requirements follow: coverage; adhesion and continuity; available fraction; partitioning and penetration where required; target-site persistence; and surface presentation. Their relative weight is set by target state and barrier condition, not by intrinsic superiority of any delivery platform. No study cited in this work measures transcuticular delivery of an antifungal from an edible coating to a quiescent *Colletotrichum* infection in intact fruit. Sustained-release performance has been characterized in vitro and against surface spoilage organisms, but not against a shielded target across an intact mango cuticle, and closing this gap requires the available fraction to be quantified at the cuticle–epidermal interface rather than at the fruit surface. Section 3 then modifies these spatial requirements by temperature, relative humidity, condensation, storage duration, handling history, and the continued cuticle deposition documented in this section.

## 3. Postharvest Storage Trajectories as Dynamic Constraints on Antifungal Delivery

### 3.1. From Static Storage Conditions to a Time-Resolved Delivery

Postharvest storage is a sequence of environments rather than a single temperature and relative humidity value. Coating application is followed by refrigerated storage, transport and holding, and in most supply chains a warmer shelf-life phase. The pathogen, the fruit surface and the delivery matrix all pass through this sequence, and each responds on its own timescale. A storage condition is therefore better treated as a point in a history than as a fixed property of the system.

The fruit interface also evolves during storage. In ‘Sun’ peach stored for 14 d at 0 °C followed by 5 d at 20 °C, cuticle mass increased from 15.1 g m^−2^ at harvest to 17.4 g m^−2^ after cold storage and 19.2 g m^−2^ after subsequent warming, while expression of cuticle-related genes was suppressed during refrigeration and recovered to varying degrees after transfer to 20 °C [56]. In mango, pre-chilling conditioning altered membrane integrity during subsequent cold storage and shelf life, indicating that the sequence of temperature exposure, rather than temperature alone, can influence postharvest responses [68]. Similarly, the delivery matrix is sensitive to the storage trajectory. Deng et al. (2018) [69] reported a water vapor permeability of 0.06 g mm m^−2^ d^−1^ Pa^−1^ for a chitosan Pickering system, compared with 0.251 g mm m^−2^ d^−1^ Pa^−1^ for the more hydrophilic chitosan–cellulose nanocrystal film. In a three-month pear storage trial at 1.7 °C and 90% RH, however, the study evaluated ripening and physiological disorders rather than fungal disease. Accordingly, release and retention measured under one set of conditions may not reflect behavior on intact fruit exposed to a different storage trajectory. The remainder of this section therefore considers whether antifungal availability remains temporally aligned with the phase at which disease risk emerges. Relative humidity is particularly relevant to hydrophilic coating matrices such as chitosan because water sorption can modify polymer-chain mobility, swelling, barrier properties, and active-compound diffusion; these effects are considered explicitly in Section 3.3.

### 3.2. Temperature Acts Unequally on Pathogen, Host Tissue and the Fruit Surface

Temperature does not act on *Colletotrichum* as a single entity. Salotti et al. (2022) [70] compiled temperature responses for mycelial growth, conidial germination, infection and sporulation across 118 studies. For the *C. gloeosporioides* clade, fitted mycelial growth extended from 4 to 40 °C with an optimum near 26 °C and germination was fitted from 9 to 38 °C, whereas species-level *C. gloeosporioides* data reported infection from 10 to 35 °C and no infection below 9 °C or above 35 °C. Sandoval-Contreras et al. (2020) [71] likewise recorded much slower growth at 13 °C than at 28 °C. Refrigeration therefore shifts these processes away from their optima without being fungicidal, and it does not displace them to the same extent, so suppression of infection during cold storage does not remove the inoculum that becomes active once fruit is warmed.

For mango, low temperature carries a host cost. Zhang et al. (2017) [68] stored Guifei mango at 5 °C for 25 d before transfer to 25 °C, and untreated fruit developed chilling injury with increased membrane leakage and lipid peroxidation. Conditioning at 12 °C for 24 h before cold storage reduced injury and preserved membrane integrity. Storage temperature is therefore bounded by pathogen suppression on one side and host tolerance on the other, and it cannot be lowered far enough to remove infection risk. The residual risk falls to the delivery system.

The fruit surface changes over the same period. Belge et al. (2019) [56] showed continued cuticle accumulation during cold storage and warming. Yang et al. (2023) [72] found that Korla pear wax became more fluid over 20 d at 25 °C, with melting onset falling from 50.6 to 42.9 °C and enthalpy from 125.5 to 86.7 kJ kg^−1^ as fluid olefins accumulated. Postharvest cuticle responses also differ among commodities and cultivars [11,73]. The interface presented to a coating is therefore dynamic in amount, composition and physical state, and the direction of that change is commodity-specific. Lee et al. (2024) [15] found that a thyme oil nanoemulsion delayed anthracnose during 30 d at 10 °C, with control fruit reaching 100% incidence by day 18 while treated fruit remained near 50% at day 30. In Shah et al. (2021) [10], a chitosan–thyme oil composite reduced natural decay over 28 d at 12 °C followed by 5 d at 25 °C, although the controlled *C. gloeosporioides* lesion assay in that study was conducted separately for 9 d at 25 °C. These results establish feasibility under refrigerated and transition conditions without establishing that any architecture is superior.

### 3.3. Relative Humidity, Pathogen Water Availability, and Interfacial Hydration

Ambient relative humidity, the water available to the pathogen and the hydration of the fruit–coating interface are related but distinct variables. In inoculated papaya at 28 °C, Sandoval-Contreras et al. (2020) [71] observed similar radial growth rates of 2.4 to 2.5 mm d^−1^ at 40%, 75% and above 85% RH, although time to visible mycelium shortened numerically with the increase in humidity. Fruit weight loss varied much more strongly across the same range, which indicates a clearer effect of ambient humidity on host water balance than on the growth rate of an established infection.

Humidity also affects both the fruit interface and the coating matrix. Tredenick and Farquhar (2021) [67] modeled water transport through cellulose-associated, aqueous-pore, and lipophilic pathways and showed that hydration develops progressively, indicating that cuticular transport properties cannot be regarded as fixed after a change in storage humidity. This effect is particularly important for chitosan because its hydrophilic polymer network strongly sorbs water. Kurek et al. [74] showed that increasing RH from 0% to 100% caused substantial swelling and water-induced plasticization of chitosan films, while carvacrol diffusion and release increased strongly with RH. Similarly, Aguirre-Loredo et al. [75] reported that chitosan films absorbed up to approximately 40% moisture at high RH; increasing moisture content increased elongation and water-vapor permeability while decreasing tensile strength, Young’s modulus, and glass-transition temperature. These observations indicate that RH changes the physical state of the chitosan matrix rather than merely altering fruit water loss.

For antifungal fruit coatings, this behavior leads to a testable delivery hypothesis. At high RH, water sorption and plasticization may increase polymer-chain mobility and facilitate diffusion of incorporated antifungal compounds, potentially increasing their short-term availability at the fruit surface. However, the same process could weaken barrier performance and accelerate active depletion if release occurs before the pathogen becomes susceptible or before quiescent infection resumes growth. Conversely, under lower RH, reduced matrix hydration may favor retention but restrict the fraction of active compound released from the coating. The net effect should therefore depend on whether humidity-induced release is temporally aligned with pathogen activation. Deng et al. (2018) [69] already showed greater humidity sensitivity of a hydrophilic chitosan–cellulose nanocrystal matrix than of the corresponding Pickering-containing system, while humidity-responsive packaging studies reported increasing cinnamaldehyde and carvacrol release with increasing RH [76,77]. Because these latter studies were not conducted with *Colletotrichum* on coated fruit, the proposed relationship between RH, chitosan hydration, antifungal release, and infection-site exposure remains a mechanistic hypothesis requiring direct validation.

### 3.4. Protection-Window Matching, Duration Tested Versus Protection Demonstrated

Storage duration, the measured endpoint, and demonstrated disease protection are distinct parameters that are often conflated. Deng et al. (2018) [69] stored pears for three months but evaluated ripening, scald, and core breakdown. Hira et al. (2022) [78] maintained ‘Kosui’ pear quality for 21 d at 20 °C without significantly reducing weight loss, while Magri et al. (2024) [79] assessed browning and antioxidant responses in fresh-cut pear for 10 d at 2 °C. Iqbal et al. (2024) [80] evaluated kiwifruit quality, decay incidence, and total bacterial counts over 30 d at 10 ± 2 °C. These storage periods therefore do not provide direct evidence of protection against anthracnose. Some studies demonstrated an anthracnose endpoint. Lee et al. (2024) [15] showed protection persisting through 30 d of continuous refrigerated storage at 10 °C, with untreated Irwin mangoes reaching 100% incidence by day 18 while fruit coated with a 2 g/100 mL thyme oil nanoemulsion showed approximately 50% incidence at day 30 and maintained a disease index below grade 1. Shah et al. (2021) [10] followed natural decay across a cold-to-warm trajectory, with the pathogen-specific inoculation test conducted separately.

The requirement is temporal matching of active antifungal persistence to that of infection biology. Active availability must be retained through the phase in which infection is constrained and must be sufficient at the interface once conditions become permissive, so a reported release profile is uninformative unless the required window is specified alongside it. Added formulation complexity does not by itself meet that requirement. Lee et al. (2024) [15] found no advantage from combining thyme oil with D-limonene over thyme oil alone in mycelial inhibition, and the combination produced higher fruit respiration.

### 3.5. Pretreatment and Application History Establish the Interface Entering Storage

Part of the interface is created before storage begins. Yang et al. (2022) [81] showed that hot water dipping of kumquat at 40 °C for 5 min delayed visible decay from 45 to 70 d and reduced decay at 90 d, despite lower total wax in treated fruit at the later samplings. The treatment rearranged epicuticular wax and changed the proportions of the major wax classes, which associates storage performance with wax organization rather than with wax amount alone. Heat and carbon dioxide shocks likewise altered peach cuticle composition and wax-to-cutin ratios measured after cold storage and shelf life [56]. Thermal history is therefore a boundary condition for the barrier that a coating must later work through.

Application method then determines the film actually present on the fruit. Wang et al. (2023) [59] compared dip, brush, conventional spray and electrostatic spray application of the same mango coating system. Dip and electrostatic methods produced more complete films, whereas brush films showed tracks, cracks and pores. After 10 d, weight loss was 10.45% with dipping and 13.97% with brushing. Nominal formulation alone therefore does not define the applied architecture, and application method belongs in any description of a delivery system.

### 3.6. Storage-to-Antifungal Coating Design Synthesis

Synthesis of Section 3 is illustrated in Figure 2. Storage conditions determine whether an antifungal coating maintains sufficient active availability when disease risk is greatest. Temperature simultaneously affects pathogen activity and fruit physiology, while relative humidity influences coating hydration, matrix swelling, and active release. The fruit surface also evolves during storage through changes in cuticle amount, composition, and physical state. Preservation performance therefore cannot be inferred from initial active loading or storage duration alone. Effective design requires retention during restrictive phases and sufficient interfacial availability once conditions become permissive. Current evidence supports this principle only indirectly, as no study has continuously quantified active concentration or release at an intact coating–cuticle interface across a realistic storage trajectory, and humidity-responsive release has mainly been demonstrated in packaging systems.

Key gaps remain in quantifying active release and spatial distribution on intact coated fruit during cold storage and subsequent warming, resolving hydration dynamics and aqueous pore adjustment in attached cuticles, and monitoring cuticular permeability longitudinally during storage. Future studies should therefore report the full temperature and humidity trajectory, transition timing, pretreatment history, coating application and film characteristics, release conditions, active concentration over time, biological endpoints, and inoculation procedures. Distinctions between wounded and intact fruit, ambient and in-package humidity, and commodity-specific chilling limits are also essential. These parameters provide the basis for comparing coating architectures according to trajectory-matched antifungal availability rather than nominal formulation or storage duration alone.

## 4. Comparative Performance of Antifungal Coating Designs

Postharvest storage imposes dynamic rather than fixed protection requirements, as temperature, relative humidity, condensation, storage duration, pretreatment, and subsequent warming can alter both the coating matrix and incorporated active. Accordingly, an architecture effective early in storage may become inadequate later. This section evaluates five coating designs for postharvest anthracnose control: monolithic or hydrogel coatings, nanoemulsion-loaded coatings, Pickering-emulsion coatings, layer-by-layer systems, and electrospun or fibrous systems. Each is assessed using common criteria, including release mechanism, active stabilization, dominant failure mode, direct fruit and pathogen evidence, storage-window compatibility, evidence strength, application feasibility, and sensory or regulatory considerations. Antifungal efficacy is distinguished from preservation outcomes such as firmness, respiration, weight loss, color, and ripening, while release or barrier measurements are not treated as direct evidence of infection-site exposure. Because studies vary in host, inoculation, storage, and formulation, these criteria identify evidence gaps without implying direct comparability among architectures. Architecture alone does not determine coating function; the chemical composition of the matrix also governs interfacial and biological behavior. Chitosan contains primary amino groups that become protonated under acidic conditions, giving it cationic and pH-dependent properties [50]. In contrast, alginate, pectin, and carboxymethylcellulose contain carboxyl groups that are predominantly anionic under many coating conditions, whereas starch and cellulose derivatives are rich in hydroxyl groups that primarily support hydrogen bonding and hydration. Proteins such as gelatin contain amino, carboxyl, and amide groups and therefore show pH-dependent amphoteric behavior [12,82]. These functional groups influence polymer–polymer association, water sorption, ionic crosslinking, adhesion, and interactions with incorporated antifungal compounds. Consequently, coatings with similar macroscopic architectures may exhibit different release and interfacial behavior because their underlying chemical functionalities differ.

### 4.1. Monolithic Hydrogels and Conventional Biopolymer Coatings

Monolithic systems provide the baseline architecture for edible antifungal coatings. In this design, an active antifungal agent is dispersed directly within a film-forming matrix such as chitosan, alginate, carboxymethylcellulose, starch, gelatin, pectin, or protein-based polymers such as zein. These materials are not interchangeable: chitosan is a cationic polysaccharide in acidic conditions, while alginate, pectin, and carboxymethylcellulose are mainly anionic, and starch is mostly neutral. As a result, their interactions with active compounds, fruit surfaces, water, and microbial cells vary. Subsequent delivery depends on hydration and diffusion through that matrix. This must be distinguished from the intrinsic antimicrobial or host-response effects of the coating matrix. Chitosan is not a chemically uniform polymer, and nominal concentration alone is insufficient to define the material used in a coating. Degree of deacetylation (DD) determines the proportion of glucosamine units bearing protonatable primary amino groups and therefore influences charge density, solubility, intermolecular interactions, film formation, and antimicrobial behavior under acidic conditions [50,83]. Molecular weight and the related degree of polymerization influence chain mobility, entanglement, solution viscosity, film structure, and diffusion of incorporated active compounds. These variables are interdependent: viscosity is not an independent molecular descriptor because it also depends on polymer concentration, solvent composition, pH, ionic strength, and measurement conditions. Likewise, particle size is relevant primarily when chitosan is used as nanoparticles, colloidal aggregates, or other particulate forms rather than as a molecularly dissolved film-forming polymer. Consequently, comparisons among nominally similar chitosan coatings should report, where available, DD, molecular weight, polymer concentration, and the physicochemical conditions under which the coating solution was prepared.

The properties of a chitosan coating are also strongly formulation-dependent. Degree of deacetylation determines the abundance of protonatable amino groups and therefore affects charge density, solubility, antimicrobial activity, polymer–active interactions, and adhesion [50,83]. Molecular weight and degree of polymerization influence chain entanglement and solution viscosity; increasing viscosity can improve film-forming capacity and coating retention but can also alter spreading, coating thickness, and diffusion of incorporated antifungal compounds. Because viscosity additionally depends on polymer concentration, pH, ionic strength, and temperature, it should not be interpreted independently of these variables. Polymer concentration similarly affects solution rheology, deposited film thickness, water uptake, mechanical properties, and gas- and water-vapor transport; excessive viscosity or thickness may improve barrier performance while limiting uniform spreading, gas exchange, or active-compound release.

The solvent and processing environment provide an additional source of variability. The acid used to dissolve chitosan determines both protonation and the counter-ion present in the dried matrix; acetic, lactic, citric, hydrochloric, and other acids can produce different chain interactions, crystallinity, mechanical behavior, and film structure [84]. Final pH controls the fraction of protonated amino groups and therefore influences solubility, antimicrobial activity, adhesion, and interactions with charged actives or fruit-surface components, whereas ionic strength modifies electrostatic interactions and polymer conformation [85]. Drying temperature and rate can alter film moisture, swelling, mechanical properties, and water-vapor transport, and these effects interact with polymer concentration [86]. Film thickness increases the diffusion path length and directly affects practical gas and vapor transmission and the time required for active compounds to migrate through the coating, although normalized permeability coefficients should be distinguished from transmission rate. When chitosan is used in nanoparticulate form, particle size and size distribution additionally influence specific surface area, colloidal stability, interfacial contact, and antifungal performance; these effects are formulation- and pathogen-dependent rather than universally proportional to decreasing particle size [87]. Consequently, on-fruit performance cannot be attributed to ‘chitosan concentration’ alone, because material grade, solution chemistry, film formation, and final coating geometry jointly determine antifungal exposure, barrier behavior, and fruit physiological response.

On artificially inoculated White Chaunsa mango, 1% chitosan containing 400 µL L^−1^ thyme oil reduced lesion diameter to 14.8 mm after nine days at 25 °C, compared with 66.25 mm in the control. The formulation also reduced naturally occurring decay during storage at 12 °C and performed comparably with prochloraz in that experiment [10]. In another mango study, 15 g L^−1^ chitosan with 0.12 g L^−1^ iturin A produced the greatest lesion suppression after 20 days at 12 °C. Lesion diameter was 69.1% lower than the control, compared with 41.8% for chitosan alone [22]. Nascimento et al. (2020), using low-molecular-weight chitosan with 64% deacetylation and a molecular weight of 1.6 × 10^4^ g mol^−1^, reported an MIC of 5 mg mL^−1^ and an MFC of 10 mg mL^−1^ against *C. gloeosporioides* [88]. Coated fresh-cut guava also showed better microbiological control under refrigeration than at 24 °C. These findings show that active loading can add disease control beyond that of the matrix alone, and that this architecture is compatible with simple dipping under the tested conditions. However, detailed chitosan descriptors such as degree of deacetylation and molecular weight were not consistently reported across the coating studies considered here, which limits direct comparison of nominally similar ‘chitosan’ treatments.

Release kinetics of active antifungals from monolithic coatings have not been adequately characterized under conditions representative of commercial storage. Consequently, whether early burst release limits sustained infection-site exposure remains unresolved. Monolithic coatings have demonstrated disease control over short- to moderate-duration storage under the specific conditions tested [10,22], but their ability to maintain inhibitory concentrations throughout longer or changing storage trajectories has not been directly quantified.

### 4.2. Nanoemulsion-Loaded Coatings

Nanoemulsion-loaded designs add a dispersed oil phase and an extra partition step to simple matrix incorporation. They add droplet-matrix partitioning to polymer diffusion, reducing essential oil volatilization and extending release relative to monolithic coatings [64,89]. In oxidized starch and pullulan films, release of the cinnamon essential oil nanoemulsion followed an initial rapid phase and sustained Fickian diffusion [89]. Das et al. (2023) [14] encapsulated *Angelica archangelica* essential oil in a chitosan nanoemulsion ranging from 53.69 to 98.63 nm, demonstrating biphasic controlled release in vitro in which the minimum inhibitory concentration (MIC) against *Botrytis cinerea* decreased from 5.5 to 2.5 g L^−1^, and maintained the quality of table grapes for 30 days at 25 ± 2 °C. Lee et al. (2024) [15] reported that a thyme oil nanoemulsion at 2 g/100 mL inhibited *C. gloeosporioides* mycelial development by 50.9% in vitro and reduced anthracnose incidence and disease index in Irwin mango during 30 days at 10 °C. Nanoemulsions therefore improve essential oil dispersion and persistence, but remain constrained by oil loading, droplet stability, polymer-network integrity, essential oil volatility, aroma, and sensory acceptability [90].

Temperature is also a critical determinant of nanoemulsion stability. Temperature changes can alter oil and surfactant solubility, interfacial properties, droplet collision frequency, and, consequently, coalescence or Ostwald ripening. In a D-limonene nanoemulsion stored at 4, 25, and 50 °C, increasing temperature altered droplet size, ζ-potential, viscosity, and turbidity and decreased the encapsulated fraction while increasing D-limonene release [91]. Thus, temperature transitions during cold storage and subsequent shelf life may modify both nanoemulsion integrity and active availability, although the magnitude of this effect is formulation-dependent. Nanoemulsions consequently have direct disease evidence under ambient and refrigerated storage, including 30-day tests. No supplied study measured infection-site concentration, so broader superiority cannot be inferred from droplet stability, release profiles, or preservation outcomes.

### 4.3. Pickering-Emulsion Coatings

Pickering systems use solid particles instead of molecular surfactants to stabilize the oil–water interface. These particles form a protective interfacial layer around essential oil droplets, which improves emulsion stability, limits droplet coalescence and diffusion-driven droplet growth through Ostwald ripening, and slows essential oil release [92]. Deng et al. (2018) [69] showed that cellulose nanocrystal (CNC) Pickering emulsion-incorporated chitosan films had significantly lower water vapor permeability than CNC-reinforced chitosan films without the Pickering architecture, at 0.06 against 0.251 g mm m^−2^ d^−1^ Pa^−1^. In the same study, CH-PCNC-coated Bartlett pears showed no senescent scald after three months of storage at 1.7 °C and 90% RH. Zhang et al. (2024) [93] confirmed sustained ginger essential oil release from a CNC-stabilized Pickering emulsion in a sodium alginate film and maintained mango firmness at 15.91 N on day 11 (*p* < 0.05). Wardana et al. (2021) [94] showed that 0.24% cellulose nanofibre stabilized chitosan and sandalwood oil Pickering emulsions without compromising antifungal activity, with the sandalwood oil-containing system inhibiting *Penicillium digitatum* spore germination by 86.24%.

Pickering coatings provide a strong passive platform for temporally controlled protection by stabilizing active compounds and modulating their release. However, most fruit-based studies still infer kinetic advantages from film-release profiles, barrier properties, and fruit-quality outcomes [69,93], rather than directly measuring active-compound availability at the infection court over time.

### 4.4. Layer-by-Layer Coatings

Layer-by-layer (LbL) assembly exploits complementary polymer charges, for example, protonated amino groups in chitosan and carboxylate groups in alginate or carboxymethylcellulose, to construct sequential polyelectrolyte layers, enabling control over composition, spatial arrangement, and thickness [82]. Hira et al. (2022) [78] applied chitosan and alginate layers to Kosui Japanese pear for 21 days at 20 °C. The five-layer treatment reduced oxygen transmission, suppressed respiration and ethylene production, and retained firmness better than monolayers. In mango stored for 20 days at 15 °C, Ali et al. (2024) [95] found that a chitosan and carboxymethyl cellulose multilayer was 224.61 µm thick, compared with 113.35 and 110.75 µm for monolayers. It also produced the lowest weight loss and physiological decay, although no disease control experiments were performed.

Active multilayer studies provide only indirect evidence for antifungal disease control. Brasil et al. (2012) [96] showed that a chitosan–pectin multilayer containing β-cyclodextrin-complexed trans-cinnamaldehyde markedly suppressed natural aerobic, psychrotrophic, yeast and mould populations in fresh-cut papaya at 4 °C, maintaining aerobic counts near 2 log CFU/g compared with approximately 8 log CFU/g in controls, and extending microbiological shelf life from less than 7 to 15 days. However, no defined pathogen was inoculated, so these findings demonstrate broad microbial spoilage control rather than pathogen-specific disease suppression. Magri et al. (2024) [79] reported reduced physiological deterioration, enzymatic browning and oxidative damage in cold-stored fresh-cut Williams pear, but did not measure microbial populations, pathogen infection, disease incidence or lesion development.

In this layer-by-layer design, repeated deposition and drying can increase the application burden. The respiratory pathway shifts reported by Hira et al. (2022) [78] also warn that barrier intensity is itself a physiological intervention. LbL systems provide modular control over coating architecture, enabling tunable permeability, gas exchange, and potentially active-agent diffusion through layer composition, charge density, and deposition sequence. However, data on release kinetics and active-compound concentrations at the infection court are lacking, which limits direct evidence that LbL coatings maintain protective doses throughout storage.

### 4.5. Electrospun and Fibrous Delivery Systems

Electrospinning produces porous nonwoven mats with high surface-to-volume ratios suitable for volatile antimicrobials or biological control organisms [97]. Gundewadi et al. (2021) [97] demonstrated polyvinyl alcohol fibers containing β-cyclodextrin complexes of thyme and betel leaf oils fixed inside boxes with inoculated sapota at 28 °C. Disease severity after six days was 8.39% with 300 ppm and 6.72% with 400 ppm, while the control reached 100%. Direct essential oil exposure caused peel burning, whereas the fibrous treatment avoided that injury. The result demonstrates disease suppression and mitigation of direct-oil phytotoxicity, but only as a packaging insert. Vázquez-González et al. (2024) [98] encapsulated *Meyerozyma caribbica* in electrospun fibers. Viability and in vitro activity were retained against *C. gloeosporioides*, but no fruit disease trial was conducted. Yuan et al. (2023) [99] likewise demonstrated in vitro *B. cinerea* inhibition and preservation of wrapped sweet cherries, not controlled disease suppression on fruit.

Core–shell or multilayer fibers could bridge burst and sustained delivery, but remain unvalidated on climacteric fruit under commercial storage. Future studies should therefore test these architectures under realistic cold-chain conditions, where high relative humidity, fruit respiration, ethylene production, and package ventilation may alter fiber hydration, essential oil partitioning, volatile retention, and antifungal dose maintenance at the fruit surface.

### 4.6. Stimuli-Responsive Architectures

Stimuli-responsive architectures may improve temporal alignment between antifungal availability and infection progression by limiting release during quiescence and increasing it when conditions become permissive for disease [1,42]. pH is a relevant trigger because *Colletotrichum* spp. can alkalinize host tissue through ammonia production, with the resulting shift associated with appressorium formation, pectate lyase production, and host cell death [46,47]. Genetic evidence supports this mechanism, as Δ*gdh2* and ammonium-transporter mutants showed impaired appressorium formation and reduced pathogenicity [48,49]. This supports coatings whose swelling, permeability, wettability, or emulsion stability changes within the infection-associated pH range [100,101]. Cellulose-based nanoparticles enabled pH-regulated oregano essential-oil release, while TEMPO-oxidized nanofibrillated cellulose/PNIPAM-acrylamide hydrogels accelerated natamycin release and delayed mango decay in packaging models [100,102]. However, the latter responded mainly to acidic conditions, leaving alkalinity-triggered release at the *Colletotrichum* infection court largely unexplored. Humidity offers another trigger because surface water favors conidial germination, infection, and sporulation [70,71]. A β-cyclodextrin MOF/carvacrol@zein film released 96.3% of carvacrol at 100% RH but only 12.0% at 43% RH by day 7 [103].

Enzyme-responsive systems could couple release more directly to biological activity. Chitosan induces host defense enzymes, although this does not demonstrate in situ degradation of a coating carrier [32,50]. *Colletotrichum* also produces laccases, cellulases, pectinases, pectate lyases, and endoglucanases during the transition from quiescence to necrotrophy [46,51]. Disruption of *LAC1* reduced virulence and extracellular enzyme activities in mango, while *Cglac4* was implicated in conidial germination and appressorium formation and was strongly suppressed by eugenol [37,45]. These findings support virulence-enzyme-activated release from degradable carriers, although enzyme-responsive packaging has not been validated for fruit anthracnose [52,53]. Ethylene is a less direct trigger. It is associated with climacteric ripening and host changes linked to quiescent infection activation, but is better regarded as an upstream indicator of necrotrophic permissiveness than as a confirmed fungal signal [1,32,104]. Direct ethylene-triggered antifungal release from edible coatings remains unproven [105]. Across these approaches, infection site validation under realistic storage trajectories remains lacking.

### 4.7. Sensory, Phytotoxicity, and Commercial Translation Constraints

Antifungal efficacy and infection-site bioavailability are necessary but not sufficient for practical translation. The effective dose must also remain compatible with fruit tolerance and consumer acceptance. This is particularly important for essential oils, because their strong aroma and potential effects on fruit flavor and taste can limit sensory acceptability even when antifungal activity is satisfactory [65]. Optical and appearance-related attributes are also relevant to marketability. Edible coatings should therefore be evaluated for changes in brightness, opacity, surface uniformity, and other visible characteristics rather than only for disease suppression and physicochemical preservation [106]. The importance of fruit tolerance is illustrated by Gundewadi et al. (2021) [97]. They observed skin-burning symptoms when sapota fruit were directly exposed to essential oils at the tested level, whereas encapsulation in electrospun nanofibers avoided direct-contact injury while maintaining anthracnose control.

Commercial applicability also depends on whether the coating architecture can be integrated into realistic postharvest handling. Wang et al. (2023) [59] showed that application method altered coating quality on mango: Dip coating and electrostatic spraying produced relatively uniform and complete films, whereas the different methods varied in transfer efficiency, film thickness, and drying requirements. Electrostatic spraying additionally reduced coating consumption and drying time and was considered suitable for continuous and automated operation. Thus, coating architecture should be evaluated together with application rate, number of deposition and drying steps, coating consumption, processing throughput, and compatibility with packinghouse operations.

Economic feasibility is an additional translational constraint, but it cannot be inferred from antifungal efficacy alone. For chitosan–essential-oil coatings, practical cost will depend on material grade and concentration, active loading, coating consumption, application method, drying requirements, and process throughput [107]. Application method can itself influence coating consumption and drying requirements, as demonstrated for mango coatings [59]. More broadly, edible-coating commercialization remains constrained by scale-up, processing, drying, and cost considerations [107]. However, most fruit-coating studies still emphasize biological efficacy and quality preservation rather than process-level economic analysis; therefore, the available evidence does not support a general conclusion that cost either enables or precludes commercial use of chitosan coatings on mango. Pilot-scale application and techno-economic assessment under packinghouse conditions are still required before commercial viability can be established.

The terms “natural,” “edible,” “food-grade,” and “commercially acceptable” should also not be treated as interchangeable. “Natural” describes material origin but does not by itself establish safety or regulatory suitability. An edible coating is intended to remain on the food and be consumed with it, while its ingredients and use levels must satisfy applicable food-safety requirements [108]. Commercial acceptability imposes additional constraints, including sensory and visual quality, processing feasibility, cost, regulatory compliance, and consumer acceptance. Consequently, advanced delivery systems should not be considered commercially promising solely because their constituent materials are natural or edible; practical translation requires simultaneous demonstration of efficacy, safety, sensory compatibility, and process feasibility. These factors are considered here as translational boundary conditions rather than as a formal commercial-feasibility framework, because comparable pilot-scale, economic, and packinghouse-performance data are not available across the coating systems reviewed.

### 4.8. Cross-Architecture Synthesis

A synthesis of the available evidence indicates that no single coating architecture is universally superior; rather, suitability depends on the biological target, storage trajectory, and required delivery behavior. Monolithic coatings provide a comparatively simple architecture and have demonstrated anthracnose control under short- to moderate-duration storage conditions [10,22], although active release at the infection site has not been quantified. Nanoemulsion-loaded coatings improve essential-oil dispersion and can extend release relative to direct matrix incorporation [64,89], with disease suppression demonstrated during storage periods of up to 30 days [14,15]; however, these studies do not establish broader superiority among architectures. Pickering systems improve emulsion stability and sustain active release [69,93,94], but whether stronger retention increases or limits infection-site availability remains unresolved. LbL assemblies permit control over coating composition, thickness, and barrier properties [78,82,95], although their repeated deposition and drying steps may increase application burden; this remains a practical consideration rather than a demonstrated commercial limitation. Electrospun systems can provide indirect or vapor-phase delivery and have reduced direct essential-oil peel injury in the tested packaging system [97], but evidence for direct-contact antifungal coatings on fruit remains limited. Stimuli-responsive systems offer a mechanistically attractive route for temporally targeted release, but direct validation against infection-site exposure remains insufficient [52,53,100,102,103]. Architecture choice should therefore be based on the specific delivery constraint and strength of available evidence rather than on assumed intrinsic advantages of any platform. These evidence gaps define the context-matching and validation priorities developed explicitly in the following section. The comparative release behavior and stage-matching implications of coating architectures are summarized in Figure 3.

**Figure 3:**
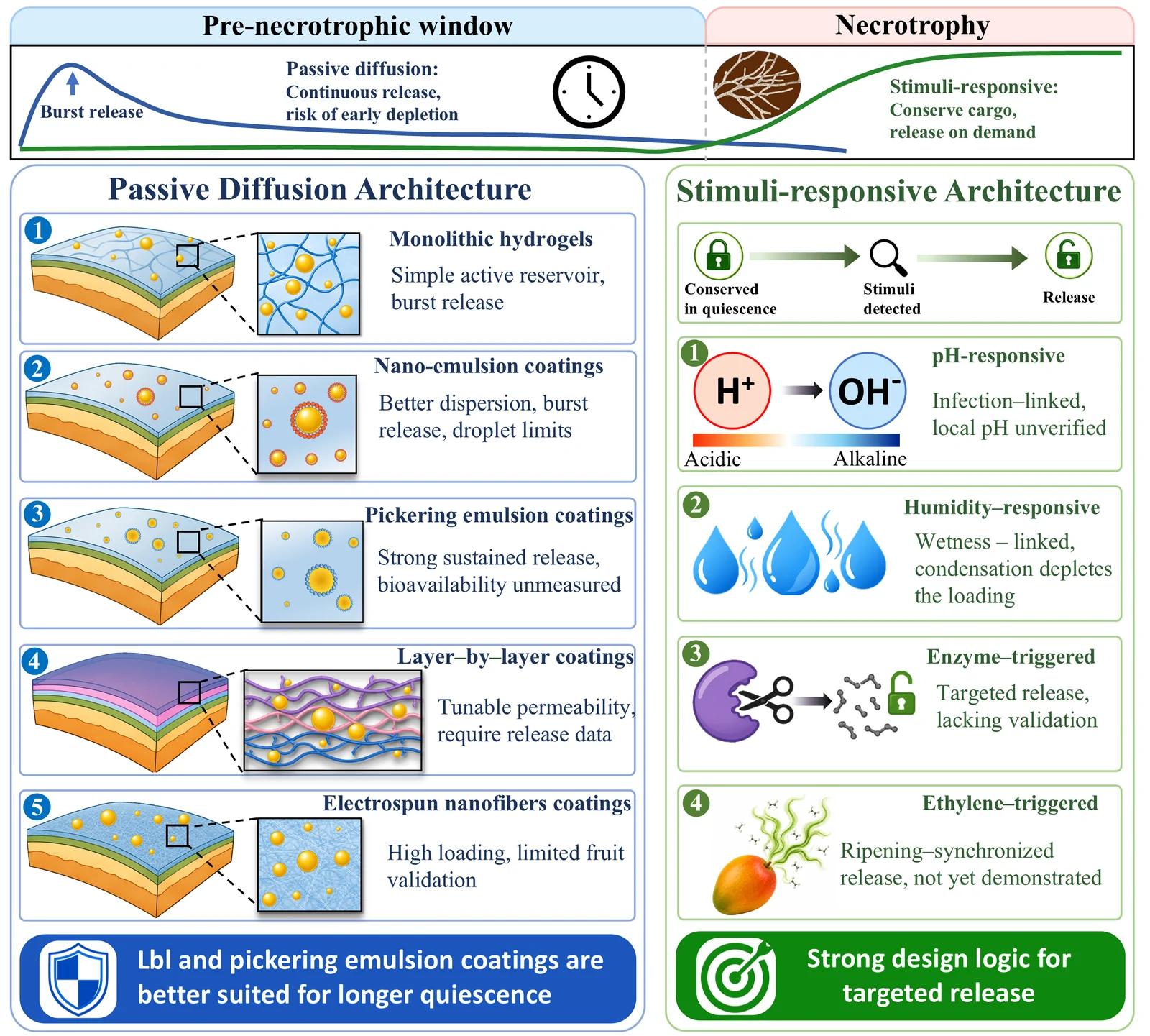
Release behavior of different antifungal edible coating architectures for postharvest anthracnose control.

## 5. Context-Matched Mechanistic Design

The biologically relevant target for an antifungal coating is not generic “slow release” but the maintenance of sufficient bioavailable active at the infection-relevant site over the required disease stage and storage period. Carrier release represents only one step, followed by transfer to the coating–fruit interface, cuticle partitioning, movement toward the infection site, and persistence against volatilization or degradation. Because cuticular transport depends on composition and temperature, bulk release measurements may not represent interfacial exposure [109]. Architecture selection should therefore be guided by where and when the active must act, the required persistence, tolerance to temperature and RH transitions, and the maximum deliverable dose within sensory and phytotoxicity limits. This framework provides a more mechanistically relevant basis for interpreting coating-release kinetics.

### 5.1. Long-Quiescence Anthracnose in Climacteric, Wax-Rich Fruit

Anthracnose in mango and avocado provides a clear case for temporal matching. *Colletotrichum* infections can remain quiescent in unripe fruit and become necrotrophic as ripening progresses [2,38,110]. The design challenge is therefore not simply to generate a high initial antifungal concentration. The coating must persist through the pre-necrotrophic period, retain an active reservoir during cold storage, maintain interfacial availability at the fruit surface, and remain functional during subsequent warming and ripening. The required profile combines reservoir persistence, limited early depletion, interfacial availability, sustained or renewed exposure near pathogen activation, and tolerance of cold-to-warm transitions. A monolithic coating may provide these functions if active–matrix affinity does not excessively reduce the transferable fraction. Nanoemulsions may improve active dispersion and surface coverage. Direct mango-anthracnose evidence shows that essential-oil nanoemulsion coatings can suppress lesion development, although this does not establish superiority over alternative architectures under matched conditions [111]. Pickering systems can improve emulsion stability and maintain antifungal performance [94], while layer-by-layer assembly allows barrier properties to be tuned through layer number and assembly conditions [112]. Stimuli-responsive designs may also be relevant where prolonged quiescence increases the risk of premature reservoir depletion. These are potential mechanistic advantages rather than evidence of universal optimality.

Temperature transitions require explicit consideration because the fruit, pathogen, cuticle, and carrier may change simultaneously. Warming alters ripening-associated host physiology and pathogen activity, while temperature can also modify molecular transport through the cuticle [2,38,109]. A formulation stable at a single refrigeration temperature may therefore exhibit a different release or transport regime during shelf life. For this scenario, experiments following the same treated fruit through cold storage and subsequent warming would provide stronger evidence than studies evaluating these phases separately.

### 5.2. Short-Window, Highly Perishable Fruit Under High Humidity

Strawberries and similarly perishable fruits impose a different set of constraints, including a short commercial life, high respiratory activity, frequent high-RH storage, and susceptibility to rapid fungal deterioration. The required profile therefore favors immediate surface protection with persistence over a comparatively short window while limiting excessive sensory loading. Extending release beyond the relevant storage period is not inherently advantageous.

Strawberry studies illustrate this distinction, although they do not provide direct evidence for anthracnose. Chitosan–pectin coatings containing lemon-essential-oil nanoemulsions combined measured essential-oil release with suppression of filamentous fungi and yeasts during strawberry storage [113]. Layer-by-layer chitosan–pectin coatings extended strawberry shelf life [114], while related work showed that LbL assembly can tune coating gas-barrier properties to fruit respiratory requirements rather than simply maximizing barrier strength [112]. A chitosan-stabilized thyme-essential-oil Pickering system also provided sustained release and preserved strawberry quality during cold storage [115]. These findings support different aspects of delivery and preservation, but they do not establish that controlled release itself caused the observed storage benefits.

The key mechanistic question is therefore whether sustained release extends biological protection or primarily limits premature active loss. A coupled mass balance that measures surface-retained active, headspace loss, interfacial availability, and disease progression under realistic RH conditions would help distinguish these mechanisms. High RH can also alter the carrier. Hydrophilic matrices may absorb water, swell, and plasticize, thereby modifying active mobility and potentially affecting adhesion or barrier properties. Release measured under dry or well-stirred model conditions may consequently differ from behavior under high-humidity package conditions. RH should therefore be treated as a design variable that can modify both the carrier structure and fruit-surface transport.

### 5.3. Wound-Dominated and Fresh-Cut Systems

Wounding bypasses the intact cuticular barrier and creates a delivery environment distinct from intact-fruit infection [38,116]. The design priority consequently shifts toward rapid local availability, adhesion, and compatibility with exposed tissue. The enzyme-responsive fresh-cut apple system of Xin et al. is considered here only as a proof of principle for biologically triggered release [117]. In that system, increased pectin methylesterase activity at the exposed cut surface promoted structural changes in the responsive Pickering carrier and enhanced thyme essential-oil release. Because cutting removes the intact cuticular barrier and alters local tissue physiology and enzyme accessibility, this mechanism cannot be extrapolated directly to natural *Colletotrichum* penetration or quiescence beneath an intact fruit cuticle. Its relevance to anthracnose is therefore conceptual rather than physiological: Analogous infection-associated signals and carrier responses would need to be demonstrated directly at intact *Colletotrichum* infection sites before such a design could be considered mechanistically validated. This distinction is particularly important when relating minimum inhibitory concentrations to fruit efficacy. In wound assays, an applied dose can be placed close to pathogen propagules without crossing the intact cuticular barrier. On intact fruit, however, the active must spread, partition at successive interfaces, persist, and reach a cuticular or subcuticular infection site. A formulation that performs strongly in a wound assay may therefore not retain the same relative performance on intact fruit, even when its intrinsic antifungal activity remains unchanged.

### 5.4. From Efficacy Gaps to a Mismatch Taxonomy

Poor translation from in vitro antifungal activity to fruit disease control is more informative when diagnosed as a specific mismatch rather than described broadly as low bioavailability. Seven operational failure modes can be distinguished. Dose mismatch occurs when nominal loading does not reflect the free or transferable active fraction because of sequestration, crystallization, degradation, or volatilization. Spatial mismatch occurs when released active fails to reach the relevant surface, cuticular, or subcuticular site. Temporal mismatch arises when burst release, delayed release, or reservoir exhaustion fails to overlap the disease-relevant period.

Environmental mismatch occurs when release or stability is characterized under conditions that do not represent cold storage, high RH, condensation, or subsequent warming. Interfacial mismatch arises when matrix–active or cuticle–active interactions impair desorption, wetting, adhesion, or partitioning across successive interfaces. Biochemical mismatch occurs when pathogen- or host-derived biochemical processes alter the coating or its expected function. For example, *Colletotrichum* produces extracellular enzymes including laccases, cellulases, pectinases, pectate lyases, and endoglucanases [46,51], which could modify chemically susceptible polymer matrices or alter triggered release, whereas bioactive polymers such as chitosan can induce host defense responses [50]. Either process could change active availability or disease progression independently of nominal loading. Direct degradation of intact antifungal coatings by *Colletotrichum* enzymes has not yet been demonstrated and should therefore be treated as a testable mechanism rather than an established failure mode. Model mismatch occurs when the experimental system removes the barrier or disease stage that the formulation is intended to overcome, as in wounded versus intact fruit, mycelial inhibition versus latent infection control, or constant-temperature assays versus cold storage followed by warming. The pH-responsive apple film of Tong et al. provides a comparatively strong evidence chain because material structure, pH-dependent release, and fruit disease outcome were evaluated within the same study [118]. However, these measurements do not by themselves demonstrate infection-site exposure or establish that triggered release was the sole cause of the observed disease reduction.

These categories are separable because they imply different formulation responses. Increasing loading may address dose mismatch but increase sensory or phytotoxic risk. Strengthening a diffusion barrier may prolong release while worsening dose or spatial mismatch. Improved wetting may enhance spatial delivery without altering carrier-release kinetics. The taxonomy is therefore diagnostic, and formulation changes should target the experimentally identified limitation rather than increase architectural complexity by default.

### 5.5. Decision Rules for Coating Design

A context-matched design framework treats architecture selection as a constraint-satisfaction problem. It begins by defining the target anatomical site and pathogen stage, reconstructing the storage trajectory, and specifying active volatility, hydrophobicity, stability, and sensory limits. The required concentration–time–space profile can then be defined, cuticular transport accounted for, and architectures selected according to the required functions. The next step is to identify the most plausible mismatch and design measurements to test that mechanism.

This logic generates testable hypotheses rather than universal rankings. In a long-quiescence pathosystem, a formulation that preserves interfacial active until warming should outperform an equally loaded system that exhausts its reservoir during cold storage. In short-window, high-RH fruit, improving surface retention may be more important than extending release beyond the commercial window. Wound-model rankings should change when repeated on intact fruit if cuticular transport is rate-limiting. For volatile systems, disease control should be more closely tied to measured exposure over time than to nominal formulation loading. These hypotheses provide a rigorous basis for interpreting current evidence without assigning unmeasured mechanisms to individual studies.

## 6. Closing the Measurement Gap for Kinetically Informed Coating Design

### 6.1. Why Current Release Assays Cannot Diagnose Mismatch

Release from edible coatings is normally quantified as cumulative migration into a simplified aqueous or food-simulant phase such as phosphate buffer, acetate buffer, ethanol or water and ethanol mixtures [14,89,119]. Ye et al. (2025) [89] identified Fickian diffusion as the dominant mechanism for cinnamon essential oil released from oxidized starch and pullulan nanoemulsion films, and Das et al. (2023) [14] reported biphasic release of *Angelica archangelica* oil from a chitosan nanoemulsion.

Four omissions matter for the argument developed here. The cuticle is absent, although it is a stratified composite of cutin, waxes, polysaccharides, and phenolics whose aliphatic wax fraction, rather than its total wax load, governs solute permeation [36,66]. Test conditions are static, although cuticular amount, composition and hydration change throughout storage [73]. Essential oil constituents are chemically labile. Volatility provides an elimination route that aqueous sink tests rarely capture [120]. A release curve therefore characterizes the coating, not the delivery.

### 6.2. Measurable Parameters and Available Tools

Each mismatch category defined in Section 5 corresponds to a parameter that can be measured with existing instrumentation (Table 2). Suitable methods exist in adjacent fields but have not been applied systematically to postharvest antifungal coatings.

Cuticular permeability is a major unresolved parameter, but it should not be treated as a fixed property of the fruit surface. Without compound-, host-, and storage-stage-specific transport information, loading density, encapsulation efficiency, and bulk release rate cannot predict whether an inhibitory concentration reaches the compartment in which quiescent infections reside [36]. Enzymatically isolated cuticles or peel membranes mounted in Franz diffusion cells can provide controlled ex vivo measurements of compound transport [121], but such measurements represent the physiological state of the cuticle at the time of sampling rather than the evolving interface of an intact fruit. Because wax deposition, composition, hydration, and physical state can change during cold storage and subsequent warming [56,72], permeability should ideally be measured using cuticles collected at multiple storage stages and then validated against transport or infection-site concentration measurements on intact fruit. Priority compounds are thymol, carvacrol, eugenol, cinnamaldehyde and defined chitosan oligomers rather than bulk chitosan, whose molecular weight strongly restricts transport.

Infection-court concentration over time is the direct measure of interfacial bioavailability. Confocal Raman microspectroscopy can resolve cuticular chemical domains at micrometer scale without requiring high-vacuum conditions [35,55]. However, application to individual essential-oil constituents would require compound-specific reference spectra, calibration, and validation of spectral sensitivity and overlap. Conventional vacuum MALDI-MSI is more problematic for volatile constituents because tissue drying and high vacuum can cause analyte loss [122]. For such compounds, ambient-pressure MSI approaches, including DESI/nano-DESI or atmospheric-pressure laser desorption methods, provide more appropriate candidates [123]; atmospheric-pressure imaging has, for example, spatially detected a monoterpene directly in fresh ginger tissue with reduced volatile loss [124]. Accordingly, spatial measurements of thymol, carvacrol, or related compounds should be validated by recovery controls and an orthogonal quantitative method such as surface extraction or headspace GC-MS before being interpreted as concentration maps.

Surface half-life separates volatilization, degradation, and tissue partitioning. Paired headspace GC-MS and surface extraction at defined intervals would yield elimination rate constants, which are absent from the reviewed literature despite volatility and instability being recognized constraints on bioavailability [65,120]. Available fraction after burst loss can be resolved by centrifugation, dialysis, and mass balance. Entrapment and loading data already exist [14,125], but neither defines the fraction surviving the burst phase.

Duration above the local minimum inhibitory concentration and temporal overlap with the pre-necrotrophic window are at present inferred from disease incidence, which is an endpoint rather than a kinetic measurement. Pathogen physiological and transcriptomic responses, such as the membrane disruption and altered metabolic signaling reported for oregano oil nanoemulsion films [90], demonstrate biological exposure but not local concentration and cannot substitute for direct measurement.

**Table 2:** Coating design mismatch parameters and available measurement tools.

Parameter	What It Represents	Mismatch Diagnosed (Section 5)	Available Tools	Closest Published Approximation
Available fraction after burst loss	Active pool remaining for sustained delivery	Dose	Centrifugation, dialysis, mass balance	Entrapment and loading efficiency [14] and formulation stability only [125]
Cuticle permeability coefficient, per compound and host	Transport rate across the fruit cuticle	Matrix–active and cuticle	Franz cell with isolated cuticle or peel disks [36,121]	Apple peel pesticide permeation [121]
Infection-court concentration over time	Local concentration at the biological target	Spatial	Confocal Raman; ambient-pressure MSI (DESI/nano-DESI or AP-LDI); surface microextraction/headspace GC-MS; validated probes	No postharvest antifungal coating equivalent identified
Duration above local MIC	Time above the inhibitory threshold at the target	Temporal	Derived from local concentration and pathogen MIC	No direct equivalent identified
Compound surface half-life	Loss by volatilization, degradation and tissue partitioning	Environmental	Headspace GC-MS with paired surface extraction	No direct equivalent identified
Overlap with the pre-necrotrophic window	Match between delivery and pathogen activation	Model	Time-resolved inoculation, local concentration mapping, disease progression, pathogen biomarkers	Inferred indirectly from disease incidence
Biochemical mismatch	Pathogen or host biochemistry alters carrier behavior or disease response	Matrix degradation/host-response modulation	Enzyme-responsive packaging [52,53]; chitosan elicitation [50]	Enzyme assays, polymer degradation/MW analysis, release kinetics, host-defense markers

### 6.3. Host and Pathogen Specificity

Any such framework must be host-specific and pathogen-specific. Cuticle composition, wax chemistry, thickness, and surface pH vary markedly among commodities, producing transport behavior that cannot be extrapolated across hosts [54]. For pH-responsive designs, the proposed alkalinization trigger should remain a testable hypothesis until local pH trajectories are measured at the infection court rather than inferred from culture systems [32,46]. Ethylene-responsive coatings for mango, avocado and banana should be described as ripening-synchronized designs, because ethylene is a host ripening signal and not a demonstrated fungal trigger. Enzyme-responsive systems require evidence that pathogen enzyme activity unlocks the matrix at the appropriate infection stage and location. Without paired in situ signal measurement, release profiling, and time-resolved disease assessment from the same formulation batch, stimuli-responsive coatings cannot be mechanistically distinguished from passive ones. Non-climacteric hosts need a separate framework, since *Botrytis cinerea* in strawberry shifts from quiescent colonization to necrotrophy as tissue senesces rather than in response to an ethylene burst [41].

### 6.4. Research Priorities

Three directions are crucial to creating the minimum dataset. The top priority is a compound-resolved, host and storage-stage-specific cuticle permeability matrix for mango, avocado, strawberry, citrus, and papaya. Permeability should be measured using isolated cuticles or peel membranes collected at defined points along the postharvest trajectory—for example, at harvest, after cold storage, and after subsequent warming—and tested in Franz diffusion cells under commodity-relevant temperature, humidity, and surface-pH conditions. These ex vivo measurements should be regarded as stage-specific transport parameters and, where possible, paired with intact-fruit concentration profiling.

The second is concentration versus time profiling from packinghouse to retail for at least three host classes under standard and temperature-abuse conditions, at 10 to 13 °C for mango where chilling injury sets the lower bound [68], and under commodity-appropriate refrigeration conditions for avocado and strawberry. Stimuli-responsive formulations should additionally be tested under the fluctuations typical of break-bulk handling, where changes in matrix mobility place trigger fidelity most at risk.

The third is a minimum reporting standard. Studies should report active loading, encapsulation efficiency, burst-release fraction, release kinetics under fruit-relevant pH and storage temperature, surface residence half-life, and paired in vitro and in situ fruit assays performed on the same formulation batch. For essential-oil formulations, botanical species, plant part, extraction method, chemotype where applicable, and the major constituent profile determined by appropriate compositional analysis should also be reported, because total essential-oil concentration alone does not adequately define the active mixture. Studies should also include inoculation timing, pathogen developmental stage, inoculum density, temperature and humidity history, coating thickness, application method, and phytotoxicity at the intended dose and release profile. For chitosan-based systems, studies should additionally report degree of deacetylation, molecular weight or viscosity grade, polymer concentration, dissolution acid and concentration, final formulation pH and ionic strength where relevant, drying conditions, and particle size/PDI for nanoparticulate formulations. Phytotoxicity assessment should include visible peel injury, browning or discoloration, and, where appropriate, quantitative indicators of tissue damage, because an antifungal exposure sufficient for disease control may not necessarily be tolerated by the fruit [97]. Release testing at pH 7.4 and 25 °C remains useful for interlaboratory comparison but cannot carry biological interpretation on its own, because it represents neither fruit-surface acidity nor the cuticular barrier nor cold-chain conditions. Adopting these three measures would convert coating development from empirical disease screening into design against a defined concentration, time and location target.

## 7. Conclusions

Existing evidence indicates that edible coatings can suppress postharvest fungal diseases, stabilize volatile or poorly soluble antifungals, modulate release, and preserve fruit quality. However, the literature also shows that high in vitro antifungal activity, extended storage duration, favorable barrier properties, or sustained release in model media do not by themselves demonstrate effective delivery to a pathogen on intact fruit. Preservation endpoints such as firmness, weight loss, respiration, and ripening likewise cannot substitute for pathogen-specific disease outcomes. This review therefore concludes that antifungal coating performance is governed by context-matched bioavailability. Effective exposure depends on the anatomical location and developmental stage of the pathogen, the physicochemical and structural properties of the fruit interface, the storage trajectory, and the retention, partitioning, release, and stability of the active compound. Across monolithic, nanoemulsion, Pickering, layer-by-layer, fibrous, and stimuli-responsive systems, no architecture is universally superior. Their value depends on which constraint dominates. Long-quiescence anthracnose requires reservoir persistence and sustained availability as fruit progresses from cold storage to ripening. Short-window, high-humidity systems emphasize immediate surface protection and resistance to humidity-induced changes. Wound and fresh-cut models present a different delivery problem because they bypass the intact cuticular barrier and, in some cases, the normal infection sequence. These differences can be categorized into dose, spatial, temporal, environmental, interfacial, biochemical and model mismatches, offering a more specific diagnostic explanation for the weak in vitro-to-fruit translation compared to the broad term “poor bioavailability”. The major unresolved gap is the measurement of active antifungal at the infection site. None of the reviewed studies directly quantifies antifungal concentration over time at the cuticle–epidermal infection site of intact fruit across a realistic storage trajectory. Compound-specific cuticle permeability, active fraction remaining after burst loss, surface residence half-life, duration above the local inhibitory concentration, and temporal overlap with pathogen activation also remain largely unmeasured. Stimuli-responsive concepts are particularly promising but require paired evidence that the proposed signal occurs in situ, activates release, and improves disease control under the same conditions. Future research should therefore prioritize host and compound-specific cuticle permeability matrices, concentration–time profiling from packinghouse to retail, spatial mapping of actives across coated fruit interfaces, and realistic cold-chain experiments incorporating temperature and humidity transitions. Standardized reporting of formulation, application, release, inoculation, and storage variables is equally important. Commercial translation also requires compatibility with existing postharvest operations. Scale-up costs for complex formulations, multi-step deposition and drying requirements, coating consumption, process throughput, and the need to modify packing or packaging lines can limit otherwise effective designs. Thus, biological and kinetic performance should ultimately be evaluated together with processing simplicity and industrial feasibility. Progress will rely less on making architectures more complex and more on creating coatings tailored to specific concentration, location, and timing needs, then experimentally confirming that these needs are met at biologically relevant sites.

## Figures and Tables

**Figure 1:**
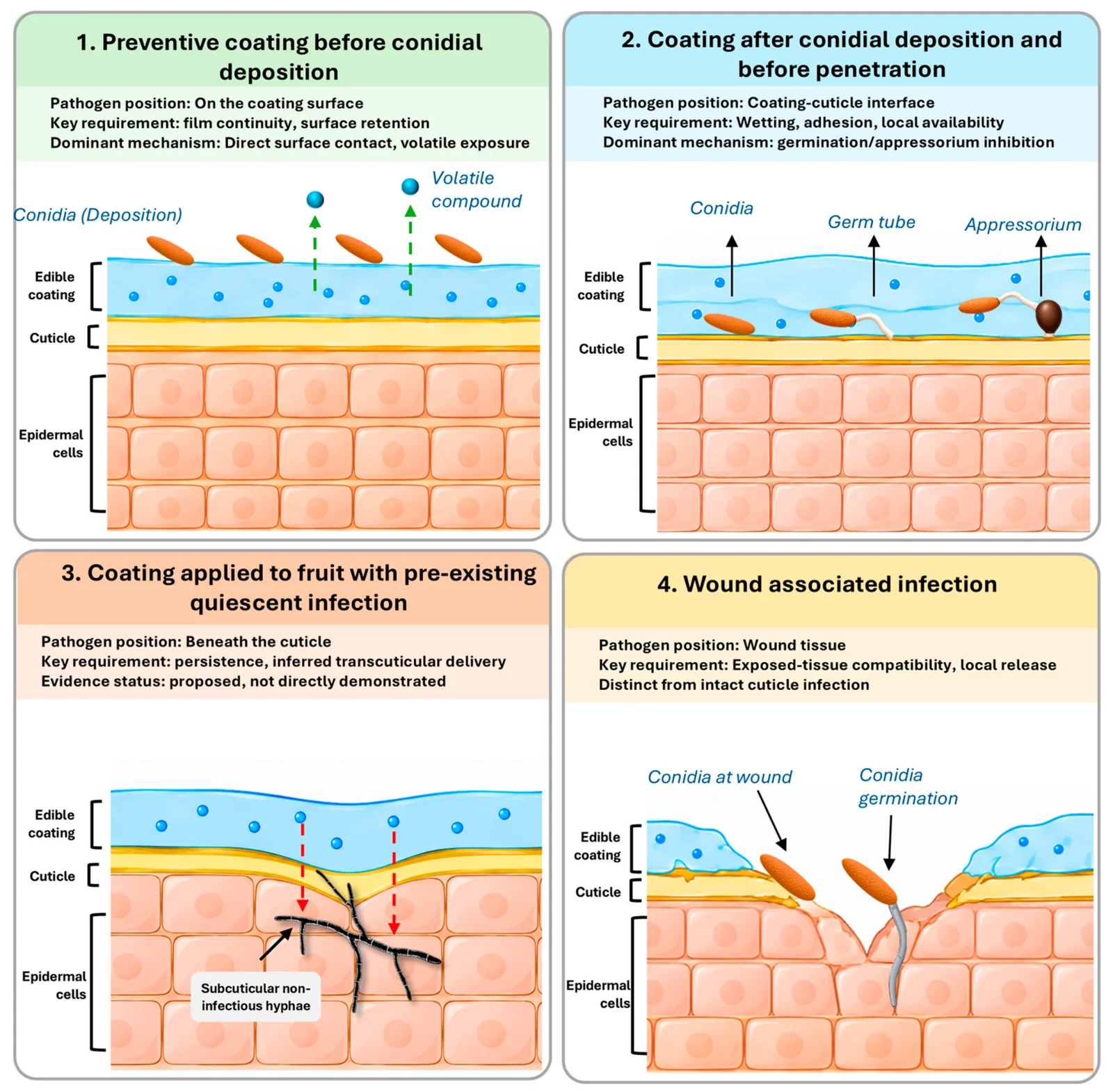
Distinct coating–pathogen exposure scenarios during *Colletotrichum* infection of fruit. The schemes illustrate (**1**) preventive coating before conidial deposition, (**2**) coating application after conidial deposition but before penetration, (**3**) coating application to fruit with a pre-existing quiescent infection beneath or within an intact cuticle, and (**4**) wound-associated infection with disruption of the cuticular barrier.

**Figure 2:**
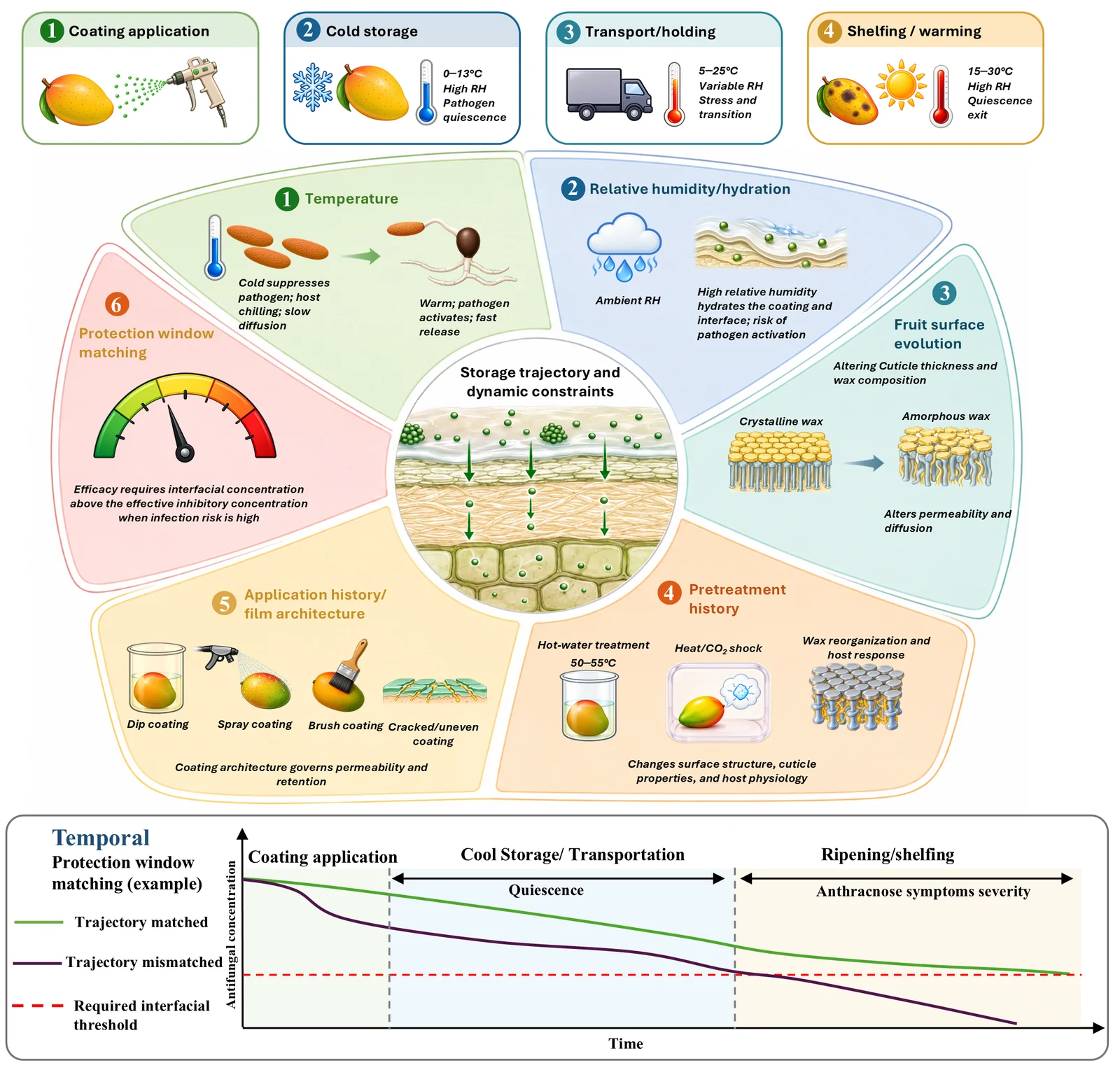
Dynamic effects of storage trajectory, temperature, environmental conditions, and pretreatment on antifungal bioavailability and temporal protection-window matching at the fruit infection site.

## Data Availability

No new data were created or analyzed in this study. Data sharing is not applicable to this article.

## Supplementary Materials

The following supporting information can be downloaded at: https://www.mdpi.com/article/10.3390/foods15193408/s1, Table S1: Complete database-specific search strings, search limits, and eligibility criteria used for literature identification.
