# Supplementary material for: From Antifungal Potency to Fruit Protection: Infection-Site Bioavailability and Delivery Constraints in Antifungal Edible Coatings
Source: Foods. 2026 Sep 24;15(19):3408. doi: 10.3390/foods15193408 (PMC13663991; doi:10.3390/foods15193408)
Supplement: Supplementary file 1 [file foods-15-03408-s001.zip › foods-4532405-supplementary.pdf]

Supplementary Materials

From Antifungal Potency to Fruit Protection: Infection-Site Bioavailability and Delivery Constraints in Antifungal Edible Coatings

Table S1- Complete database-specific search strings, search limits, and eligibility criteria used for literature identification

Database	Search	Exact search string
Web of Science Core Collection	W1: Core coating–anthracnose evidence	`TS=((Colletotrichum OR anthracnose) AND (fruit* OR postharvest) AND ("edible coating*" OR "antifungal coating*" OR chitosan OR "essential oil*" OR nanoemulsion* OR "Pickering emulsion*" OR "layer-by-layer" OR electrospun*))`
Web of Science Core Collection	W2: Infection site/cuticle/interface	`TS=((Colletotrichum OR anthracnose OR fruit*) AND ("fruit cuticle" OR cuticle* OR "epicuticular wax" OR microcrack* OR lenticel* OR "quiescent infection" OR appressor* OR adhesion OR wettability OR partition* OR permeab* OR transcuticular) AND (coating* OR antifungal* OR delivery OR release))`
Web of Science Core Collection	W3: Delivery and measurement evidence	`TS=(("controlled release" OR "sustained release" OR "stimuli-responsive" OR "pH-responsive" OR "humidity-responsive" OR "enzyme-responsive" OR "mass spectrometry imaging" OR Raman OR "Franz diffusion cell") AND (fruit* OR cuticle* OR food) AND (coating* OR packaging OR antifungal* OR volatile*))`
Scopus	S1: Core coating–anthracnose evidence	`TITLE-ABS-KEY((colletotrichum OR anthracnose) AND (fruit* OR postharvest) AND ("edible coating*" OR "antifungal coating*" OR chitosan OR "essential oil*" OR nanoemulsion* OR "Pickering emulsion*" OR "layer-by-layer" OR electrospun*))`
Scopus	S2: Infection site/cuticle/interface	`TITLE-ABS-KEY((colletotrichum OR anthracnose OR fruit*) AND ("fruit cuticle" OR cuticle* OR "epicuticular wax" OR microcrack* OR lenticel* OR "quiescent infection" OR appressor* OR adhesion OR wettability OR partition* OR permeab* OR transcuticular) AND (coating* OR antifungal* OR delivery OR release))`
Scopus	S3: Delivery and measurement evidence	`TITLE-ABS-KEY(("controlled release" OR "sustained release" OR "stimuli-responsive" OR "pH-responsive" OR "humidity-responsive" OR "enzyme-responsive" OR "mass spectrometry imaging" OR Raman OR "Franz diffusion cell") AND (fruit* OR cuticle* OR food) AND (coating* OR packaging OR antifungal* OR volatile*))`
Google Scholar	G1	`"Colletotrichum" anthracnose "edible coating" fruit`
Google Scholar	G2	`"Colletotrichum" chitosan "essential oil" fruit coating`
Google Scholar	G3	`"quiescent infection" Colletotrichum fruit cuticle`
Google Scholar	G4	`"fruit cuticle" coating antifungal transport permeability`
Google Scholar	G5	`"controlled release" fruit coating nanoemulsion OR "Pickering emulsion"`
Google Scholar	G6	`"stimuli-responsive" fruit coating antifungal release`
Google Scholar	G7	`"Franz diffusion cell" fruit peel cuticle`

Google Scholar	G8	`"mass spectrometry imaging" volatile plant food`

Note: Search limits include a final search date of 24 July, 2026, with no restriction on lower publication years. The search focused on English-language, peer-reviewed journal articles, reviews, and peer-reviewed early-access articles. No proximity operators were employed. Truncation (*) was used in Web of Science and Scopus where specified. Google Scholar served as a supplementary search and citation-tracking resource rather than the main exhaustive database.
